# Insights into Mitochondrial Rearrangements and Selection in Accipitrid Mitogenomes, with New Data on *Haliastur indus* and *Accipiter badius poliopsis*

**DOI:** 10.3390/genes15111439

**Published:** 2024-11-07

**Authors:** Jumaporn Sonongbua, Thanyapat Thong, Thitipong Panthum, Trifan Budi, Worapong Singchat, Ekaphan Kraichak, Aingorn Chaiyes, Narongrit Muangmai, Prateep Duengkae, Ratiwan Sitdhibutr, Chaiyan Kasorndorkbua, Kornsorn Srikulnath

**Affiliations:** 1 Animal Genomics and Bioresource Research Unit (AGB Research Unit), Faculty of Science, Kasetsart University, Bangkok 10900, Thailand; jumaporn.s@ku.th (J.S.); typ.thong@gmail.com (T.T.); thitipong.pa@ku.th (T.P.); trifan.bu@ku.th (T.B.); worapong.si@ku.th (W.S.); ekaphan.k@ku.th (E.K.); chaiyes.stou@gmail.com (A.C.); prateep.du@ku.ac.th (P.D.); 2 Interdisciplinary Graduate Program in Bioscience, Faculty of Science, Kasetsart University, Bangkok 10900, Thailand; 3 Faculty of Interdisciplinary Studies, Khon Kaen University, Nong Khai Campus, Nong Khai 43000, Thailand; 4 Special Research Unit for Wildlife Genomics (SRUWG), Department of Forest Biology, Faculty of Forestry, Kasetsart University, Bangkok 10900, Thailand; 5 Department of Botany, Faculty of Science, Kasetsart University, Bangkok 10900, Thailand; 6 The International Undergraduate Program in Bioscience and Technology, Faculty of Science, Kasetsart University, Bangkok 10900, Thailand; 7 Department of Fishery Biology, Faculty of Fisheries, Kasetsart University, Bangkok 10900, Thailand; ffisnrm@ku.ac.th; 8 Raptor Rehabilitation Unit, Kasetsart University Veterinary Teaching Hospital, Kamphaengsaen Campus, Nakhon Pathom 73140, Thailand; ratibutr@gmail.com; 9 Department of Pathology, Faculty of Veterinary Medicine, Kasetsart University, Bangkok 10900, Thailand; 10 Laboratory of Raptor Research and Conservation Medicine, Faculty of Veterinary Medicine, Kasetsart University, Bangkok 10900, Thailand; 11 Laboratory of Animal Cytogenetics and Comparative Genomics (ACCG), Department of Genetics, Faculty of Science, Kasetsart University, Bangkok 10900, Thailand; 12 Biodiversity Center Kasetsart University (BDCKU), Kasetsart University, Bangkok 10900, Thailand

**Keywords:** raptors, phylogeny, evolution, duplication, concerted evolution

## Abstract

**Background/Objectives:** Accipitridae mitogenomes exhibit unique structural variations, including duplicated control regions (CRs) that undergo gradual degeneration into pseudo-CRs, revealing a complex evolutionary landscape. However, annotation of this characteristic in a subset of accipitrid genomes is lacking. Due to the taxonomic diversity of Accipitridae and the presence of understudied species, comprehensive mitogenomic studies are essential. This study sought to expand and investigate the evolutionary characteristics of Accipitridae mitogenomes. **Methods:** A comparative analysis was conducted using the newly acquired complete mitogenomes of *Haliastur indus* and *Accipiter badius poliopsis* along with 22 available accipitrid mitogenomes. Codon usage, selective pressure, phylogenetic relationships, and structural variations were comparatively analyzed. **Results:** Accipitrid mitogenomes showed a strong AT bias with adenine preference. All protein-coding genes (PCGs) were under purifying selection. The *ATP8* gene exhibited relaxed purifying selection on codon usage patterns and showed high genetic variation. Selection for the *ATP8* gene was specific to certain clades of accipitrids. Gene order re-examination revealed both non-degenerate CRs and highly degenerate CR2 fragments in the Accipitridae family. Non-degenerate CRs were found in early diverging species, such as *Elanus caeruleus* and *Pernis ptilorhynchus orientalis*, while more recent lineages had highly degenerate CR2 fragments with missing conserved element. Repeat motifs and sequence variations were observed in the functional CR. **Conclusions:** These findings suggest that the *ATP8* gene reflects metabolic adaptations, while CRs indicate potential diversification of these accipitrid species. This study provides valuable insights into mitochondrial genome evolution within the Accipitridae family.

## 1. Introduction

The most common avian mitochondrial genome consists of 37 genes with one control region, which is formed by rearranging *ND6* and *tRNA^Glu^* from the gene order of the common vertebrate mitogenome [1]. A single tandem duplication of a fragment between the *ND5* and *tRNA^Phe^* genes, likely followed by subsequent degeneration of the duplicated elements, has been identified in most avian lineages [2,3,4,5,6,7]. However, many avian mitogenomes contain duplicated regions that were previously underestimated and have not been annotated in genomic studies [2,5,8,9]. This has affected our ability to predict the evolution and mechanisms of duplication of avian mitogenomes. In Accipitriformes, diurnal raptors exhibit diverse mitogenomic gene orders based on their taxonomic classification. These range from the fully duplicated order in Sagittaridae (GO-FD type) to the less reduced order in Pandionidae (GO-II), and advanced degeneration with decay of the second control region (CR) in Accipitridae (GO-IV) [9]. Interestingly, the representatives of Accipitridae contain two independently evolved CRs in different lineages. This differs from other families in Accipitriformes and from closely related raptor orders, such as new world vultures in Cathartiformes and nocturnal raptors in Strigiformes, in which the two CRs have undergone concerted evolution [9]. A larger number of substitutions accumulated in CR2 sequences, located between the *tRNA^Glu^* and *tRNA^Phe^* genes, than in CR1, located between the *tRNA^Thr^* and *tRNA^Pro^* genes, particularly in Accipitrinae, *Gyps fulvus*, and *Bustustur indicus*. Many repetitive DNA sequences were found in several CR2 sequences, which suggests that CR1 had been subjected to stronger selection than CR2 [9].

Accipitrids (family Accipitridae and order Accipitriformes), which include eagles, hawks, kites, and related species, are diurnal raptors with diverse body shapes and plumage patterns. They are the most diverse clade of Accipitriformes, exhibiting variations in feeding behavior and talon size [10,11]. Despite their similar morphologies, large variations exist among the subfamilies within Accipitridae [10]. Diurnal raptors are characterized by extreme flight capability, high morphological diversity, widespread migratory behavior, and worldwide distribution in all terrestrial habitats [12]. Their physical characteristics are shaped by adaptations to diverse habitats, with most migrations occurring during the daylight. This suggests that the mitogenome is crucial for extreme flight capability, since its regulation is essential for the function of complexes involved in oxidative phosphorylation, which is vital for ATP production and maintenance of metabolic homeostasis [13]. A substantial association has been reported between the presence of additional CR sequences and longevity across a wide phylogenetic range of birds [14]. Two mechanisms have been proposed for the duplication of CR increasing lifespan, namely: (1) by protecting cells from the loss of mitochondrial function due to age-related deletions; or (2) by increasing the flexibility of the mitochondrial response to environmental changes [14]. However, the relationship between mitogenome and CR duplications and the oxidative phosphorylation potential of accipitrids still remains unclear, due to the limited availability of data on annotated duplicated CR sequences [9,14]. A robust understanding of the evolutionary adaptations and phylogenetic relationships of mitogenome, including the duplication of CR, would be required to delineate the foundational information on diurnal raptors. This study aimed to investigate the current mitogenome structure of 22 accipitrid species and of two more species, *H. indus* and *A. badius poliopsis*, totaling 24 species across different subfamilies of Accipitridae. Taking advantage of their sequence similarity and gene order rearrangement, the hypothesis that all CR1 and CR2 sequences of accipitrids evolved independently was tested based on their different mutation rates and varying degrees of degeneration. This may be related to the mitogenome structure in connection with other segments of the mitogenome. The complete mitogenomes of *H. indus* and *A. badius poliopsis* were sequenced, and codon usage, selective pressure, phylogenetic relationships, and structural variations analyzed comparatively. This would provide a diverse dataset of accipitrid mitogenomes, contributing to our understanding of phylogenetic lineage radiation.

## 2. Materials and Methods

### 2.1. Sample Collection and DNA Extraction

The female brahminy kite (*H. indus*, Boddaert, 1783) used in this study was found with an injured wing in Samut Prakarn, Thailand (13°36′2″ N 100°35′48″ E) in March 2014, while the female shikra (*A. badius poliopsis*, Hume, 1874) in this study was collected from an orphan nestling in Nakhon Ratchasima, Thailand (14°58′20.1″ N 102°05′11.2″ E) in May 2020. Both *H. indus* and *A. badius poliopsis* were collected live, each identified using A Photographic Guide to the Raptors of Thailand [15]. Shikra has recently been proposed to be re-classified to genus *Tachyspiza* [16]. Blood samples were collected from the right jugular vein using a 23G 1-inch needle (Terumo, Tokyo, Japan) during veterinary care; both the birds were submitted to the Kasetsart University Raptor Rehabilitation Unit (KURU) and eventually released into the wild. All animal care and experimental procedures were approved by the Animal Experiment Committee of Kasetsart University, Thailand (Approval No. ACKU64-VET-002 and ACKU64-VET-052) and conducted in accordance with the Regulations on Animal Experiments at Kasetsart University and the ARRIVE guidelines (https://arriveguidelines.org, accessed on 8 January 2024). Genomic DNA was extracted from the blood specimens following the standard salting-out protocol [17]. Total genomic DNA was quantified using a NanoDrop 2000 Spectrophotometer (Thermo Fisher Scientific, Wilmington, DE, USA), and the quality of intact DNA was examined using 1% agarose gel electrophoresis.

### 2.2. Mitogenome Assembly and Annotation

Next-generation sequencing was performed on 100 ng/µL DNA samples using an Illumina NovaseqTM 6000 (2  ×  150 bp paired-end) with 15× coverage at Novogene Co., Ltd. (Singapore). High-quality reads for *A. badius poliopsis* were assembled and annotated using MitoZ v3.3 [18] with manual inspections to compare the DNA sequences with reference mitogenomic sequences. For *H. indus*, Illumina resequencing was performed twice, and the combined raw reads were used for de novo assembly of a mitochondrial backbone with GetOrganelle v1.7.4.1 (dependencies: Bowtie2, SPAdes, BLAST) [19]. The assembly was completed by amplicon sequencing of the control regions using Sanger and Nanopore long-read technology, followed by polishing and integration into the final mitogenome with Geneious Assembler v2025.0.3. (See Supplementary Material for details). The tRNAscan-SE web server v.2.0 was used to verify the RNA genes [20]. Mitogenomic sequences were deposited in the National Center for Biotechnology Information (NCBI) database (https://www.ncbi.nlm.nih.gov, accessed on 17 November 2025 and 2 August 2022) under accession numbers OP133375.2 for *H. indus* and LC721527.1 for *A. badius poliopsis*. The genome structure of mitochondrial DNA was visualized using OGDRAW 1.3.1 [21]. Three conserved regions, namely the terminal-associated sequence (TAS), central conserved domain, and conserved sequence blocks (CSB), were identified in both the CRs by recognizing sequences similar to those in other vertebrates [22,23,24]. Tandem repeat sequences, including the motif, length of repeats, and copy number in the CR region, were investigated using the Tandem Repeats Finder 4.09 program [25].

### 2.3. Comparative Sequence Analysis

Complete mitogenome sequences of 22 accipitrids (four species from Circinae, seven species from Accipitrinae, eight species from Buteonninae, and one species each from Aquilinae, Aegypiinae, Circaetinae, Perninae, and Elaninae), and of *Pandion haliaetus* from the family Pandionidae were retrieved from NCBI on May 10, 2023 (Table S1). Nucleotide constitution and skewness (bias toward certain nucleotides) were assessed using DAMBE7 [26]. AT and GC skew were calculated using the formulae AT = [(A − T)/(A + T)] and GC = [(G − C)/(G + C)] [27].

### 2.4. Codon Usage and Selective Pressures Across Protein-Coding Genes

Codon usage parameters included relative synonymous codon usage (RSCU), guanine-cytosine content at the first and second codon base positions (GC12), guanine-cytosine content at the third codon base position (GC3), and the effective number of codons (ENc). Analysis of RSCU was performed using the R package “cubar” [28], whereas the frequency of GC at different positions of the codon and ENc were calculated using DAMBE7 [26]. RSCU is the ratio between the observed frequency of a synonymous codon and the expected frequency of the codon when all codons are used evenly for that amino acid. Thus, RSCU directly indicated the deviation of synonymous codon usage from their even usage. RSCU > 1 indicated codon usage preference, RSCU < 1 indicated a less frequent usage of the codon, and RSCU = 1 indicated no bias in codon usage [29]. Neutrality plot analyses using GC12 and GC3 were conducted to assess the extent of influence of mutation pressure and natural selection on the pattern of codon usage [30]. ENc was used to measure the degree of codon bias, which correlated negatively with codon usage bias, with values ranging from 20 to 60. When ENc = 20, there was an absolute bias toward synonymous codons, whereas ENc = 60 indicated neutral codon usage (i.e., even usage of all codons) [31]. The ENc-GC3s plot was analyzed to assess whether codon usage in the given genes was primarily driven by mutation or influenced by other factors such as selection. Points lying around the expected curve indicate a predominant role of mutation, while points significantly departing below the curve suggest the involvement of selection. The P2 index [32] estimated the efficiency of codon–anticodon interactions, indicating the accuracy of the translational machinery of genes without any preferred codon set information. P2 was calculated using the formula (WWC + SSU)/(WWY + SSY), where W = A or U, S = C or G, and Y = C or U. P2 > 0.5 indicated the existence of translational selection in the given coding sequence.

### 2.5. Signature of Selection Analysis

The non-synonymous (K_a_) to synonymous (K_s_) substitution rates ratio was used to assess evolutionary pressure on protein-coding genes (PCGs). A value of K_a_/K_s_ ratio > 1 indicated positive selection, while K_a_/K_s_ ratio < 1 suggested purifying selection. At neutral evolution, K_a_/K_s_ ratio was equal to 1 [33]. The mean genetic distance between the annotated PCGs of the studied mitogenomes was calculated using the Kimura-2-parameter (K2P) substitution model in MEGA 11 [34], and nucleotide diversity was estimated using the R package “pegas” [35].

### 2.6. Phylogenetic Analyses and Molecular Dating

Twelve concatenated mitochondrial PCGs (excluding the *ND6* sequence located in the light chain) were aligned independently using MAFFT v7.490 [36]. ModelFinder was used to select the best-fit substitution model according to the Bayesian Information Criterion (BIC) [37]. The best-fit models for PCGs and control region datasets were GTR+F+I+G4 and TVM+F+G4, respectively, where GTR represents the general time reversible model, TVM denotes the transversion model, F signifies the use of empirical frequencies, I indicates invariant sites, and G represents the discrete γ distribution. Phylogenetic trees were reconstructed using maximum likelihood (ML) with 10,000 ultrafast bootstrap replicates in IQTREE-1.6.12 [38,39] and Bayesian inference (BI) with Markov chain Monte Carlo (MCMC) runs of 2,000,000 generations, sampling trees every 1000 generations, with the first 25% discarded as burn-in in MrBayes 3.2.6 [40] through the CIPRES Science Gateway, under the best-fit model GTR+F+I+G4. The osprey (*P. haliaetus*) of the family Pandionidae was used as an outgroup for both analyses. Phylogenetic trees were edited and visualized using iTOL [41]. The divergence time was estimated using 12 PCGs with BEAST2 on ACCESS v.2.7.8 [42]. Three calibration points were modeled as priors using a lognormal distribution, including the split between *Haliaeetus* and *Milvus* (9.6 ± 0.15 million years ago (MYA; mean ± standard deviation)), that between *Milvus* and *Buteo* (12.3 ± 0.15 MYA), and that between *Pandion* and other Acciprids (37.8 ± 0.05 MYA) [43]. The GTR model was used along with a lognormal relaxed clock algorithm (Yule speciation process) [44]. Markov chain Monte Carlo simulations were run for 10 million iterations, and the convergence was verified using Tracer v1.7.2 [45]. TreeAnnotator v.2.7.4 was used to produce a phylogenetic tree with maximum clade credibility [42].

### 2.7. Analysis of Structural Variation in Control Region

Two non-coding sequences located between the *tRNA^Thr^* and *tRNA^Pro^* genes (designated as CR1) and the *tRNA^Glu^* and *tRNA^Phe^* genes (designated as CR2) were identified to analyze the structure of the CR and assess their relationships with phylogenetic diversity. The conserved sequence elements within the CR were identified using MAFFT v7.490 [36]. Pairwise nucleotide identities between CR1 and CR2 were calculated. Variations in the structure of the CR were compared across 25 raptor species (24 Accipitridae species and one from Pandionidae). The effects of chimeric (homogenized/non-homogenized) sections on the CR1 and CR2 datasets were evaluated using a neighbor-joining tree constructed with Geneious Tree Builder and the Tamura-Nei model. Patterns of different duplications and degeneration of the CR in mitochondrial gene orders were mapped onto a phylogenetic tree generated from the 12 PCGs. Additionally, a collection of DNA sequence motifs in both CR1 and CR2 was discovered using the Multiple Expectation maximization for Motif Elicitation (MEME) suite [46]. The nucleotide sequences of conserved sequence elements within the CR were aligned using ProGraphMSA+TR [47] and compared.

## 3. Results

### 3.1. Two New Accipitrid Mitogenomes

**Figure 1:**
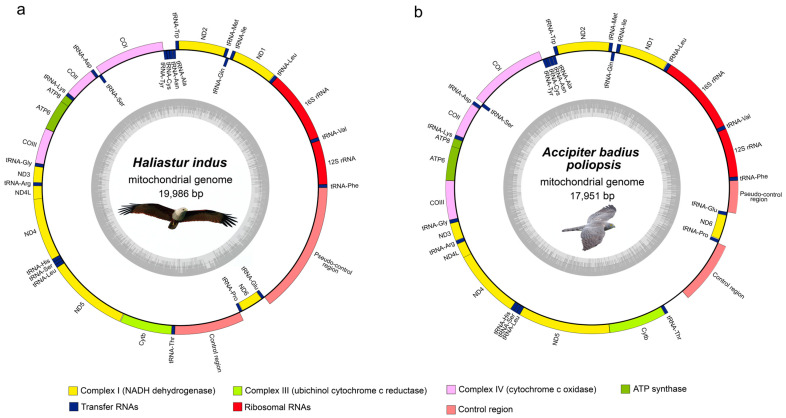
Circular maps of (**a**) *H. indus* and (**b**) *A. badius poliopsis* mitochondrial genomes.

The complete mitogenomes of *H. indus* and *A. badius poliopsis* consisted of closed circular DNA molecules with sizes of 19,986 and 17,951 bp, respectively (Figure 1). They shared a similar gene arrangement and content, containing a typical set of 37 mitochondrial genes, namely 13 PCGs, 22 tRNA genes, and two rRNA genes, and a putative CR. Eight tRNAs genes and *ND6* were encoded on the light chain (L), whereas 12 PCGs (*COI-III*, *ND1-5*, *ND4L*, *Cytb*, *ATP6*, and *ATP8*), 14 tRNA genes, two rRNA (12S rRNA and 16S rRNA) genes, and CR were encoded on the heavy chain (H) (Table S2). The standard start codon ATN was used in the PCGs of *H. indus* and *A. badius poliopsis*, except for *COI* and *ND5* in *H. indus*, which used GTG as the start codon. Three complete stop codons (TAA, TAG, and AGG) and one incomplete terminal codon were used in these two accipitrids. The TAA codon was the most frequently used in their PCGs, followed by AGG in *ND1* and *COI*, an incomplete T in *ND4* and *COIII*, and TAG in *ND2*. A variation in the stop codon between *H. indus* and *A. badius poliopsis* was found in *ND5* and *ND6*. Nucleotide composition of the *H. indus* mitogenome was A 30.6%, T 24.5%, C 30.6%, and G 14.3% while that of *A. badius poliopsis* was 30.7%, 25.0%, 31.3%, and 13.0%, respectively. The same AT-biased nucleotide composition, observed in the other mitogenomes of Accipitridae family, was shared in these mitogenomes (Table S1). The AT and GC skew values were 0.1107 and −0.3642 in *H. indus* and 0.1032 and −0.4139 in *A. badius poliopsis*.

The first non-coding region, located between the *tRNA^Thr^* and *tRNA^Pro^* genes in *H. indus* (1533 bp) and *A. badius poliopsis* (1739 bp), was assumed to be the putative control region. Three functional domains were identified in the putative control regions of *H. indus* and *A. badius poliopsis*. These included: (1) the ETAS domain, which consisted of the conserved sequence Poly-C, extended terminal-associated sequences (ETAS), and CSB1-like; (2) the Central domain, which consisted of the conserved sequences F-box, E-box, D-box, C-box, CSBa, CSBb, and Bird box; and (3) the CSB domain, which contained the conserved sequence CSB1. Interestingly, duplication of CSBb and Bird box sequences was found within the central domain of both species, although a variable number of tandem repeats (VNTR) were detected in the CSB domain of only *H. indus*. By contrast, the second non-coding region, located between the *tRNA^Glu^* and *tRNA^Phe^* genes, which is the location of the CR in typical avian mitochondrial genomes, lacked any conserved sequence. Only VNTRs of varying lengths and nucleotide repeat numbers between *H. indus* and *A. badius poliopsis* were identified (Figure S1). This non-coding region was identified as the pseudo-control region (CR2).

### 3.2. Pattern of Codon Usage in PCGs of Accipitrids

The preferred codons for each amino acid were identified using RSCU values. A concordance was observed in the predominant codon families between *H. indus* and *A. badius poliopsis*. Codons encoding leucine (Leu) was the most prevalent (Figure S2a). A similar codon usage bias was observed in the RSCU values of most codon families (Figure S2b). The similarity in codon usage patterns among the 24 accipitrid mitogenomes analyzed is shown in a heatmap based on RSCU values (Figure S3). The highest RSCU values were obtained for CUA (Leu) and CGA (Arg), whereas the lowest were for CCG (Pro), GCG (Ala), UCG (Ser), and ACG (Thr). This trend was also observed for nucleotide composition at the third codon position. Among the 30 frequently used codons, 14 ended in A and 16 ended in C. Among the less frequently used codons, 16 ended in U and 14 ended in G. The coding sequences of these accipitrids were abundant in A-ended and C-ended codons.

### 3.3. Evidence of Selective Pressures Across Protein-Coding Genes

**Figure 2:**
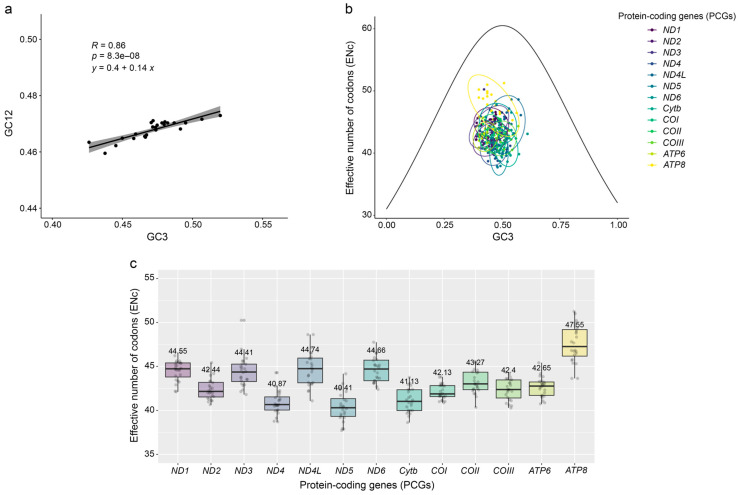
Codon analysis results of 13 protein-coding genes (PCGs) from 24 species within the Accipitridae family: (**a**) neutrality plot; (**b**) effective number of codons (ENc) plot; and (**c**) box plot of ENc of 24 species in each PCGs.

The neutrality plot showed that GC3 values ranged from 42.6 to 52.0%, with a slope value of 0.14 (Figure 2a). Additionally, the ENc graph revealed that all PCGs in the accipitrid mitogenomes fell below the expected ENc curve (Figure 2b). A comparison of ENc values across PCGs revealed that *ATP8* had a relatively high average ENc value of 47.55, whereas the other PCGs had average ENc values less than 45 (Figure 2c).

### 3.4. Translational Efficiency in the Mitogenome of Accipitrids

A preference for WWC and SSC over WWU and SSU was indicated by the P2 index, suggesting the selection for cytosine (C) over uracil (U) at the third codon position. A mean P2 index greater than 0.5 was observed in four PCGs, namely *ND2*, *ND3*, *COI*, and *COII* (Figure S4a). The P2 index of *ATP8* gene exhibited greater variability than that of other genes. Considering the P2 index in each raptor species, that of *ATP8* gene was found to vary according to the subfamily. In Accipitrinae and four primitive subfamilies (Perninae, Circaetinae, Aegypiinae, and Aquilinae), the *ATP8* gene’s P2 index was mostly above 0.5, whereas in the Elaninae, Buteoninae, and Circinae subfamilies, it was mostly below 0.5 (Figure S4b and Table S3). Additionally, the *ND3* gene in the Buteoninae subfamily had a P2 index exceeding 0.5.

### 3.5. Evolution of Accipitrid Mitogenomes

K_a_/K_s_ analysis showed that *ATP8* had the highest average non-synonymous substitution rates (K_a_), whereas the average synonymous substitution rates (K_s_) were relatively similar among PCGs. Consequently, *ATP8* exhibited the highest K_a_/K_s_ ratio (0.322), while the K_a_/K_s_ ratios of the other PCGs ranged from 0.024 to 0.201 (Table 1 and Figure S5a–c). Evolutionary distance estimation among the 24 accipitrid species revealed that *ATP8* had the largest K2P genetic distance values, followed by *ND3* and *ND6*, whereas *COI* had the lowest value (Figure S5d).

### 3.6. Phylogenetic Relationships and Divergence Times

Phylogenetic analyses using 12 concatenated PCGs revealed consistent topologies across the accipitrid species for both ML and BI methods (Figures S6 and S7). *Circaetus pectoralis* and *G. fulvus* were clustered together, whereas *Aquila nipalensis* was placed with Accipitrinae and Buteoninae. The phylogenetic position of *H. indus* suggested a sister clade relationship with *Milvus migrans*. Point estimates for the Accipitridae ages ranged from 18 to 33 MYA (Figure S8)

### 3.7. Structural Variation in the CRs of Accipitrid Mitogenomes

**Figure 3:**
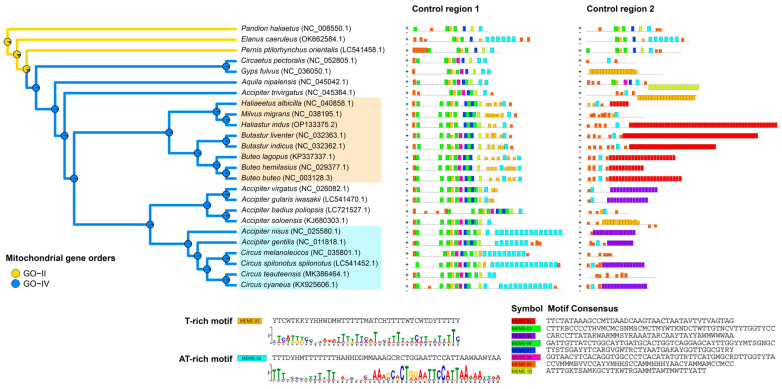
Mitochondrial gene order and collections of DNA sequence motifs found in both CR1 and CR2 regions. Mitochondrial gene orders (GO-II and GO-IV) mapped onto a phylogenetic tree based on 12 protein-coding genes (PCGs) are shown on the left, whereas the distribution of conserved motifs within control region 1 (CR1) and control region 2 (CR2/pseudo-CR) is shown on the right. Colored boxes indicate conserved motifs identified by MEME. Plus (+) and minus (−) symbols denote forward and reverse strand orientations, respectively. Lineages associated with the T-rich (MEME-05) and AT-rich (MEME-02) motifs are highlighted in orange and light blue boxes on the phylogenetic tree.

The structures of CR in *H. indus* and *A. badius poliopsis* were compared to those of 22 accipitrid species and the Ospreys (*P. haliaetus*), revealing different structural patterns, such as the loss of CR or loss of domains. CR1 consistently contained all three conserved domains (ETAS, Central, and CSB) in all the raptors examined. By contrast, CR2 possessed these domains only in the basal clade species (*P. ptilorhynchus orientalis* and *E. caeruleus*) and *P. haliaetus* (Table 2). The neighbor-joining tree of CR1 and CR2 sequences revealed that CR1 and CR2 were mostly separated in the accipitrids, except in *P. ptilorhynchus orientalis*, *E. caeruleus*, and *P. haliaetus*, where they clustered together (Figure S9). This finding was consistent with the high pairwise identity between CR1 and CR2 in these species, with values of 88.9%, 78.4%, and 87.3%, respectively (Table 2). Additionally, a study of enriched motifs revealed distinct patterns in CR1 and CR2. The motifs corresponding to the conserved sequences are displayed in CR1 (MEME-03 overlaps with parts of the F-box, D-box, and CSBA; MEME-05 overlaps with parts of the D-box and C-box; MEME-06 corresponds to the bird-box region; and MEME-07 corresponds to the CSBb region). By contrast, CR2 primarily contained repeat motifs throughout the region. Notably, two types of repeat motifs near the end of CR1 were specific to certain raptor lineages, namely an AT-rich motif in the subfamily Circinae and a T-rich motif in the subfamily Buteoninae (Figure 3).

## 4. Discussion

Using 24 mitogenomes, structural variation of mitogenomes and a robust phylogeny derived from PCGs of accipitrid birds were obtained, revealing phylogenetic relationships that were consistent with previous studies [43,48,49]. The recovered placement of *C. pectoralis*, *G. fulvus*, and *A. nipalensis* supported the previous topologies [43,48]. The clade of Accipiter species clustered with *Circus*, *Buteo*, *Butastur*, *Milvus*, *Haliastur*, and *Haliaeetus* species, supporting the observations of *Accipiter* polyphyly by Oatley et al. [49] and Mindell et al. [43]. The origin of accipitrids was dated to the late Paleogene (33.0 MYA; CI 30.1–36.0). Accipitrinae and Buteoninae diverged during the Miocene approximately 24 and 20 MYA, respectively. The mitogenomes of *H. indus* and *A. badius poliopsis* were highly congruent with those of other accipitrids, exhibiting negligible A-skew (ranging from 0.0568 to 0.1532) and significant C-skew (ranging from −0.4239 to −0.3642). This suggests an asymmetry in the nucleotide composition between the two strands, with one strand rich in A and C and the other rich in T and G in the accipitrid lineage.

### 4.1. Various Degeneration Patterns in CR2 of Accipitrids

The phylogenetic distribution of mitogenomic gene orders and reconstruction of ancestral states indicate that the common ancestor of Accipitriformes had a fully duplicated gene order, known as GO-FD based on the classification of mitogenome structure [9], which was the plesiomorphic state. In this order, the gene segments for *Cytb*, *tRNA^Thr^*, *tRNA^Pro^*, *ND6*, *tRNA^Glu^*, and the CR were tandemly duplicated. The feature was maintained in *Sagittarius serpentarius* (Sagittariidae, Accipitriformes), which is representative of the most basal family in the order Accipitriformes. Tandemly duplicated regions have also been found in the mitogenomes of other animals, such as bats, alligators, and ark clams [50,51,52], but their functions and associations with energy production capacity may differ. Further biochemical and physiological studies would be required to clarify this relationship. Concerted evolution of CRs can make these fragments identical, thereby facilitating common regulation. Processes initiated from both CR copies can increase the number of genomic and transcript copies per mitochondrion, suggesting increased mitochondrial energy production capacity [5,6,14,53,54,55]. *P. haliaetus* (Pandionidae, Accipitriformes) evolved into GO-II type by losing the first copies of *tRNA^Pro^*, *ND6*, and *tRNA^Glu^*, along with the *Cytb* pseudogene and the second copies of *tRNA^Thr^* while retaining the non-degenerate CR. By contrast, within Accipitridae, the most recent family of Accipitriformes, representative mitogenomes degenerated to the GO-IV type, with CR2 degenerated [9]. Re-examination of the mitogenomes of *P. ptilorhynchus orientalis* (LC541458.1) and *E. caeruleus* (OK662584.1) showed that their duplicate CRs had similar sequences within the species, with non-degenerate CR2s. This differed from *P. ptilorhynchus* (MK043029.1), as described previously [9]. CR1 and CR2 remain paralogous, as observed in *P. haliaetus* from the Pandionidae family [9]. This suggests that the GO-II type, characterized by concerted evolution, was present in the early diverging lineages of the Accipitridae family (Figure 3). Remarkably, sequence analyses and phylogenetic relationships of CRs in other GO-IV accessions revealed that the divergence between orthologous copies from different species (CR1 or CR2 among species) was lower than that between paralogous copies (CR1 and CR2 within species). This suggests independent evolution of the two CRs in recent accipitrid lineages. A larger number of substitutions accumulated in CR2 sequences than in CR1s, suggesting a stronger selection of CR1, which replaced the degenerate CR2 in many accipitrids. CR2 tended to degenerate due to more substitutions. Most CR2s gained repetitive DNA sequences, especially at their 3′ end. Molecular dating had indicated independent evolution at least 40 MYA [9]. The low frequency of duplicated CR homogenization may have resulted from the D-loop region not constituting a strong barrier for the replication fork. This decreased the probability of nascent strand exposure, and consequently, the recombination of mitogenome strands [56,57,58]. Additionally, duplicated regions tended to be eliminated owing to the prolonged replication time and increased energy required to synthesize a longer genome [9]. Substantial selection may have occurred in Strigiformes, Cathartiformes, Sagittaridae, Pandionidae, and the early diverging lineages of Accipitridae, since they possess a full second CR copy.

**Figure 4:**
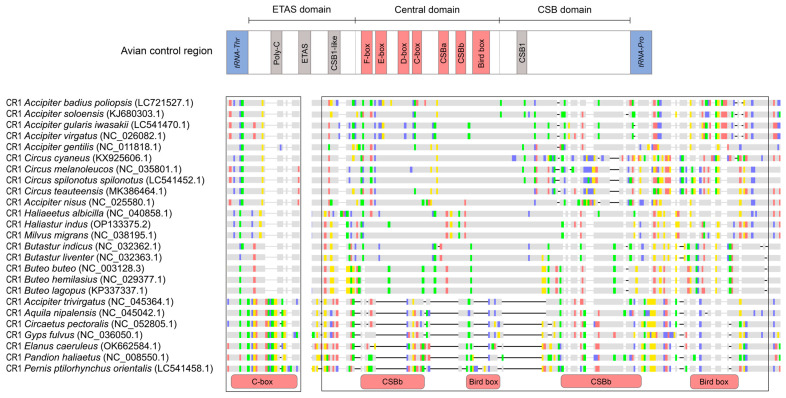
Nucleotide sequence alignment of the conserved sequence element in CR1. The upper schematic represents the avian control region, with conserved sequence boxes (F, E, D, C, and bird), CSBa, and CSBb in the central domain, shown in red. Conserved sequence elements in other domains are shown in grey. The boxed region highlights sequence variations with corresponding annotations. Colored blocks represent nucleotide differences (A, C, G, and T), whereas grey blocks indicate identical nucleotides.

Interestingly, the nucleotide sequence alignment revealed that accipitrids in recently diverging lineages (subfamilies Buteoninae, Accipitrinae, and Circinae) had different central domain sequences than early diverging species. CSBb and Bird-box variations were homogenized in specific species lineages, whereas others did not evolve simultaneously. An unequal evolutionary pattern was observed in the central domain of CR1, where CSBb and Bird boxes were duplicated, except in *A. trivirgatus* (Figure 4). Additionally, the C-box was partially deleted in recently diverging lineages (subfamilies Buteoninae, Accipitrinae, and Circinae), except in *A. trivirgatus*, and corresponds to the Replication Fork Barrier (RFB) region that halts replication [3,57]. Dominant AT-rich and T-rich repeat motifs in Circinae, *A. gentilis*, *A. nisus*, and Buteoninae were identified in different phylogenetic clades, even from different genera. This suggests targeted selection pressures on specific CR1 regions, potentially leading to functional divergence between these accipitrid lineages, with variable fragments showing no sign of homogenization.

### 4.2. Large Variation in Codon Bias of the ATP8 Gene in Accipitrid Birds

Previous studies had shown that mitochondrial oxidative phosphorylation modulates the high aerobic capacity of bird flight muscles. Thus, the mitogenome may be a significant target for natural selection in birds exposed to cold temperatures and low oxygen pressure. In this study, the codon family for leucine (Leu) was identified as the most prevalent. The 24 accipitrid mitogenomes showed similar codon usage biases with those of other Cathartiformes and Accipitriformes birds [59]. Translational efficiency indicated a high selection potential of codon bias for *ND2*, *ND3*, *COI*, and *COII* genes (P2 > 0.5), whereas *ATP8* showed a large P2 variation. The large number of codons used in the translation of the *ATP8* gene suggests weaker selection constraints on replication speed, translation efficiency, and accuracy, which affected gene expression and energy metabolism in accipitrid birds. The neutrality plot showed a GC3 slope of 0.14, indicating 14% mutation pressure and 86% evolutionary selection pressure. Selection during the translation process suggests that codon usage bias in the mitogenome of accipitrid raptors results from translational selection rather than from mutation pressure. Selection of the translational process of PCGs, particularly *ATP8*, is specific to different subfamilies of accipitrid birds. The wide range of ENc values for *ATP8* (43.65 to 51.26) may indicate different levels of selection, but under purifying selection with K_a_/K_s_ ratio < 1. This suggests that the relaxed purifying selection of the *ATP8* gene is associated with high variation in codon usage patterns and genetic variation in accipitrid birds, which may have similar functional repertoire sizes despite sequence divergence and codon bias. Many animal lineages exhibit highly variable mitochondrial DNA (mtDNA) owing to their high mutation rates, allowing the analysis of evolutionary events over short timescales. Slightly deleterious mutations contribute to polymorphism, but are removed by purifying selection over time, preventing substantial inter-species variation. Purifying selection is crucial for mtDNA evolution, since mtDNA contains essential genes for mitochondrial oxidative phosphorylation and adaptation to oxygen-starved or high-metabolism lifestyles [60]. Mutations in *ATP* and *ND* genes affect proton translocation efficiency, balancing heat generation and *ATP* synthesis [61,62,63]. The *ATP* and *ND* genes were selected simultaneously [62,64]. However, whether the large variation in codon bias of the *ATP8* gene and the degeneration of CR2 in accipitrid birds correlate with DNA replication and functional aspects, or are merely coincidental, still remains unclear. This could also be related to flight capability, high morphological diversity, widespread migratory behavior, and worldwide distribution in all terrestrial habitats. Comparative functional mitogenomics of *ATP8* and both CRs would be required in future to gain insights into the mitogenome evolution in accipitrid birds.

## 5. Conclusions

Re-examination of the existing mitogenomes and the newly acquired complete mitogenomes revealed the presence of both GO-II and GO-IV advanced degenerate arrangements in the family Accipitridae. The GO-II arrangement, characterized by paralogs of duplicated CRs, was found in early divergent species, such as *E. caeruleus* and *P. ptilorhynchus orientalis*. The latter lineages were identified using the GO-IV arrangement in which one of the CRs lacked specific conserved elements. While CR2 tends to degenerate, CR1 is subject to stronger selection, likely due to its role in replication and transcription. Notably, the variation pattern of repeat motifs and sequence variations in the central domain of CR1 in certain lineages suggests potential functional diversification. In addition, variable codon usage for *ATP8* indicated adaptive changes in energy metabolism. However, the exact relationship between these factors and the observed morphological and ecological diversity of accipitrids would require further investigation using functional mitogenomics.

## Figures and Tables

**Table 1:** Genetic distance and non-synonymous/synonymous (K_a_/K_s_) values for 13 PCGs across 24 accipitrid mitogenomes.

Gene	Substitution Model	K2P Genetic Distance	Nucleotide Diversity (π)	K_a_	K_s_	K_a_/K_s_ Ratio
*ND1*	HKY + G + I	0.15964	0.14064	0.03637	0.52465	0.06933
*ND2*	TN93 + G + I	0.17310	0.15034	0.07250	0.45150	0.16057
*ND3*	HKY + G	0.19368	0.15984	0.09606	0.47800	0.20095
*ND4*	HKY + G + I	0.15160	0.13486	0.04622	0.44860	0.10304
*ND4L*	HKY + G	0.16407	0.14272	0.05371	0.47831	0.11229
*ND5*	GTR + G + I	0.15378	0.13623	0.05898	0.44092	0.13376
*ND6*	HKY + G + I	0.18927	0.16228	0.06739	0.56474	0.11932
*COI*	GTR + G + I	0.12199	0.10999	0.01112	0.46565	0.02389
*COII*	TN93 + G + I	0.12287	0.10987	0.02295	0.43864	0.05233
*COIII*	TN93 + G + I	0.12388	0.11193	0.02549	0.43445	0.05868
*Cytb*	GTR + G + I	0.13520	0.12148	0.02928	0.46563	0.06288
*ATP6*	HKY + G + I	0.15753	0.13885	0.05554	0.43703	0.12708
*ATP8*	TN93 + G + I	0.24230	0.19855	0.15808	0.49053	0.32226

**Table 2:** The structure of control regions in the mitogenome of raptors in the Accipitridae and Pandionidae families.

Lineages	Species	CR1			CR2/Pseudo-CR			Pairwise Identity (%)
		ETAS	Central	CSB	ETAS	Central	CSB	
Family Accipitridae								
Circinae	*Circus cyaneus*	🗸	🗸	🗸	-	-	-	18.5
	*Circus teauteensis*	🗸	🗸	🗸	-	-	-	27.2
	*Circus spilonotus spilonotus*	🗸	🗸	🗸	-	-	-	17.5
	*Circus melanoleucos*	🗸	🗸	🗸	-	-	-	12.0
Accipitrinae	*Accipiter gentilis*	🗸	🗸	🗸	-	-	-	18.4
	*Accipiter nisus*	🗸	🗸	🗸	-	-	-	18.7
	*Accipiter soloensis*	🗸	🗸	🗸	-	-	-	29.5
	*A. badius poliopsis*	🗸	🗸	🗸	-	-	-	15.8
	*Accipiter gularis iwasakii*	🗸	🗸	🗸	-	-	-	33.5
	*Accipiter virgatus*	🗸	🗸	🗸	-	-	-	32.1
Buteonninae	*Buteo buteo*	🗸	🗸	🗸	-	-	-	36.5
	*Buteo hemilasius*	🗸	🗸	🗸	-	-	-	14.1
	*Buteo lagopus*	🗸	🗸	🗸	-	-	-	28.5
	*B. indicus*	🗸	🗸	🗸	-	-	-	17.4
	*Butastur liventer*	🗸	🗸	🗸	-	-	-	15.9
	*M. migrans*	🗸	🗸	🗸	-	-	-	23.0
	*H. indus*	🗸	🗸	🗸	-	-	-	38.5
	*Haliaeetus albicilla*	🗸	🗸	🗸	-	-	-	24.3
Accipitrinae	*Accipiter trivirgatus*	🗸	🗸	🗸	-	-	-	27.4
Aquilinae	*A. nipalensis*	🗸	🗸	🗸	-	-	-	36.5
Aegypiinae	*G. fulvus*	🗸	🗸	🗸	-	-	-	30.0
Circaetinae	*C. pectoralis*	🗸	🗸	🗸	-	-	-	24.6
Perninae	*P. ptilorhynchus orientalis*	🗸	🗸	🗸	🗸	🗸	🗸	88.9
Elaninae	*E. caeruleus*	🗸	🗸	🗸	🗸	🗸	🗸	78.4
Family Pandionidae								
	*P. haliaetus*	🗸	🗸	🗸	🗸	🗸	🗸	87.3

## Data Availability

Mitogenomic sequences generated from this study were deposited in the National Center for Biotechnology Information (NCBI) database (https://www.ncbi.nlm.nih.gov, accessed on 17 November 2025 and 2 August 2022) under accession numbers OP133375.2 for *H. indus* and LC721527.1 for *A. badius poliopsis*.

## Supplementary Materials

The following supporting information can be downloaded at: https://www.mdpi.com/article/10.3390/genes15111439/s1, Figure S1: Structure of mitochondrial control region of *H. indus* and *A. badius poliopsis*; Figure S2: Codon usage and Relative Synonymous Codon Usage (RSCU) in *H. indus* and *A. badius poliopsis*; Figure S3: Heat map of Relative Synonymous Codon Usage of raptor species in the family Accipitridae; Figure S4: Translational efficiency of 13 protein-coding genes (PCGs) among 24 species within the Accipitridae family; Figure S5: K2P genetic distance and the K_a_/K_s_ ratio of 13 PCGs among 24 species within the Accipitridae family; Figure S6: Phylogenetic relationships within the Accipitridae family based on maximum likelihood; Figure S7: Phylogenetic relationships within the Accipitridae family based on Bayesian inference; Figure S8: Divergence time estimates within the Accipitridae family; Figure S9: Phylogenetic tree of CR1 and CR2 inferred by neighbor-joining method; Table S1: Nucleotide composition, GC frequency, and skewness of mitogenomes in the family Accipitridae; Table S2: Mitochondrial genome organization of *H. indus* and *A. badius poliopsis*; Table S3: Translational efficiency of mitogenomes in the family Accipitridae; File S1: Detailed methods for the mitogenome sequencing and assembly of *H. indus*.
