# Correction: Sonongbua et al. Insights into Mitochondrial Rearrangements and Selection in Accipitrid Mitogenomes, with New Data on *Haliastur indus* and *Accipiter badius poliopsis*. *Genes* 2024, *15*, 1439

**DOI:** 10.3390/genes17010085

**Published:** 2026-01-13

**Authors:** Jumaporn Sonongbua, Thanyapat Thong, Thitipong Panthum, Trifan Budi, Worapong Singchat, Ekaphan Kraichak, Aingorn Chaiyes, Narongrit Muangmai, Prateep Duengkae, Ratiwan Sitdhibutr, Chaiyan Kasorndorkbua, Kornsorn Srikulnath

**Affiliations:** 1 Animal Genomics and Bioresource Research Unit (AGB Research Unit), Faculty of Science, Kasetsart University, Bangkok 10900, Thailand; jumaporn.s@ku.th (J.S.); typ.thong@gmail.com (T.T.); thitipong.pa@ku.th (T.P.); trifan.bu@ku.th (T.B.); worapong.si@ku.th (W.S.); ekaphan.k@ku.th (E.K.); chaiyes.stou@gmail.com (A.C.); prateep.du@ku.ac.th (P.D.); 2 Interdisciplinary Graduate Program in Bioscience, Faculty of Science, Kasetsart University, Bangkok 10900, Thailand; 3 Faculty of Interdisciplinary Studies, Khon Kaen University, Nong Khai Campus, Nong Khai 43000, Thailand; 4 Special Research Unit for Wildlife Genomics (SRUWG), Department of Forest Biology, Faculty of Forestry, Kasetsart University, Bangkok 10900, Thailand; 5 Department of Botany, Faculty of Science, Kasetsart University, Bangkok 10900, Thailand; 6 The International Undergraduate Program in Bioscience and Technology, Faculty of Science, Kasetsart University, Bangkok 10900, Thailand; 7 Department of Fishery Biology, Faculty of Fisheries, Kasetsart University, Bangkok 10900, Thailand; ffisnrm@ku.ac.th; 8 Raptor Rehabilitation Unit, Kasetsart University Veterinary Teaching Hospital, Kamphaengsaen Campus, Nakhon Pathom 73140, Thailand; ratibutr@gmail.com; 9 Department of Pathology, Faculty of Veterinary Medicine, Kasetsart University, Bangkok 10900, Thailand; 10 Laboratory of Raptor Research and Conservation Medicine, Faculty of Veterinary Medicine, Kasetsart University, Bangkok 10900, Thailand; 11 Laboratory of Animal Cytogenetics and Comparative Genomics (ACCG), Department of Genetics, Faculty of Science, Kasetsart University, Bangkok 10900, Thailand; 12 Biodiversity Center Kasetsart University (BDCKU), Kasetsart University, Bangkok 10900, Thailand

The authors would like to correct a mitochondrial genome assembly error identified in *Haliastur indus* in their original paper [1]. After reassembling and validating the corrected mitogenome sequence, downstream analyses relying on this sequence were updated. Consequently, the figures, tables, and references have been revised. The authors apologize for any inconvenience caused. The corrections introduced minor changes and improve the accuracy of the analyses, while the core scientific conclusions remain consistent with the original publication. This correction was approved by the Academic Editor. The original publication has also been updated.

## 1. Modified Main Text

### 1.1. Abstract

Change in the *ND3* selection result due to an alignment-related dataset error. All statements referring to positive selection in *ND3* were removed.

“Most protein-coding genes (PCGs) were under purifying selection except for *ND3*, which underwent positive selection.” was corrected to “All protein-coding genes (PCGs) were under purifying selection.”.

“Selection for *ATP8* and *ND3* genes was specific to certain clades of accipitrids.” was corrected to “Selection for the *ATP8* gene was specific to certain clades of accipitrids.”.

“These findings suggest that *ATP8* and *ND3* genes reflect metabolic adaptations, while CRs indicate potential diversification of these accipitrid species.” was corrected to “These findings suggest that the *ATP8* gene reflects metabolic adaptations, while CRs indicate potential diversification of these accipitrid species.”.

### 1.2. Materials and Methods

Section 2.2, Paragraph 1: Change due to correction of the workflow description.

“High-quality reads were assembled and annotated using MitoZ v3.3 [18] with manual inspections to compare the DNA sequences with reference mitogenomic sequences.” was corrected to “High-quality reads for *A. badius poliopsis* were assembled and annotated using MitoZ v3.3 [18] with manual inspections to compare the DNA sequences with reference mitogenomic sequences.”.

“For *H. indus*, MitoZ generated two contigs that were assembled into a single scaffolded sequence using Geneious Prime v2022.1.1. Reference-based annotation was performed using the grey-faced buzzard (*B. indicus*) mitogenome (AB830616).” was corrected to “For *H. indus*, Illumina resequencing was performed twice, and the combined raw reads were used for de novo assembly of a mitochondrial backbone with GetOrganelle v1.7.4.1 (dependencies: Bowtie2, SPAdes, BLAST) [19]. The assembly was completed by amplicon sequencing of the control regions using Sanger and Nanopore long-read technology, followed by polishing and integration into the final mitogenome with Geneious Assembler v2025.0.3. (See Supplementary Material for details).”.

“Mitogenomic sequences were deposited in the National Center for Biotechnology Information (NCBI) database (https://www.ncbi.nlm.nih.gov, accessed on 27 July 2022 and 2 August 2022) under accession numbers OP133375 for *H. indus* and LC721527 for *A. badius poliopsis*.” was corrected to “Mitogenomic sequences were deposited in the National Center for Biotechnology Information (NCBI) database (https://www.ncbi.nlm.nih.gov, accessed on 17 November 2025 and 2 August 2022) under accession numbers OP133375.2 for *H. indus* and LC721527.1 for *A. badius poliopsis*.”.

Section 2.5, Paragraph 1: Change due to correction of the method description.

“The mean genetic distance between the annotated PCGs of the studied mitogenomes was calculated using the Kimura-2-parameter (K2P) substitution model in the DnaSP software v6.12.03 [33].” was corrected to “The mean genetic distance between the annotated PCGs of the studied mitogenomes was calculated using the Kimura-2-parameter (K2P) substitution model in MEGA 11 [34], and nucleotide diversity was estimated using the R package “pegas” [35].”

Section 2.6, Paragraph 1: Change due to correction of the method description.

“The proportion of different sites (*p*-distance) between these sequences was calculated using MEGA 11 [40].” was removed.

“The divergence time was estimated using 12 PCGs with BEAST2 on XSEDE v.2.7.3 [41].” was corrected to “The divergence time was estimated using 12 PCGs with BEAST2 on ACCESS v.2.7.8 [42].”.

“Markov chain Monte Carlo simulations were run for 10 million iterations, and the convergence was verified using LogCombiner v. 1.8.4 [44].” was corrected to “Markov chain Monte Carlo simulations were run for 10 million iterations, and the convergence was verified using Tracer v1.7.2 [45].”.

“TreeAnnotator v. 1.8.4 was used to produce a phylogenetic tree with maximum clade credibility.” was corrected to “TreeAnnotator v.2.7.4 was used to produce a phylogenetic tree with maximum clade credibility [42].”.

### 1.3. Results

Section 3.1: Change due to correction of the mitogenome sequence.

Paragraph 1

“The complete mitogenomes of *H. indus* and *A. badius poliopsis* consisted of closed circular DNA molecules with sizes of 19,055 and 17,951 bp, respectively (Figure 1).” was corrected to “The complete mitogenomes of *H. indus* and *A. badius poliopsis* consisted of closed circular DNA molecules with sizes of 19,986 and 17,951 bp, respectively (Figure 1).”.

“The TAA codon was the most frequently used in their PCGs, followed by AGG in *ND1* and *COI*, and an incomplete T in *ND4* and *COIII*.” was corrected to “The TAA codon was the most frequently used in their PCGs, followed by AGG in *ND1* and *COI*, an incomplete T in *ND4* and *COIII*, and TAG in *ND2*.”.

“A variation in the stop codon between *H. indus* and *A. badius poliopsis* was found in *ND2* and *ND6*.” was corrected to “A variation in the stop codon between *H. indus* and *A. badius poliopsis* was found in *ND5* and *ND6*.”.

“Nucleotide composition of the *H. indus* mitogenome was A 30.7%, T 24.9%, C 31.0%, and G 13.4% while that of *A. badius poliopsis* was 30.7%, 25.0%, 31.3%, and 13.0%, respectively.” was corrected to “Nucleotide composition of the *H. indus* mitogenome was A 30.6%, T 24.5%, C 30.6%, and G 14.3% while that of *A. badius poliopsis* was 30.7%, 25.0%, 31.3%, and 13.0%, respectively.”.

“The AT and GC skew values were 0.1041 and −0.3959 in *H. indus* and 0.1032 and −0.4139 in *A. badius poliopsis*.” was corrected to “The AT and GC skew values were 0.1107 and −0.3642 in *H. indus* and 0.1032 and −0.4139 in *A. badius poliopsis*.”.

Paragraph 2

“The first non-coding region, located between the *tRNA^Thr^* and *tRNA^Pro^* genes in *H. indus* (1544 bp) and *A. badius poliopsis* (1739 bp), was assumed to be the putative control region.” was corrected to “The first non-coding region, located between the *tRNA^Thr^* and *tRNA^Pro^* genes in *H. indus* (1533 bp) and *A. badius poliopsis* (1739 bp), was assumed to be the putative control region.”.

Section 3.2, Paragraph 1: Change due to correction of the mitogenome sequence and cleaned datasets.

“Codons encoding leucine (Leu), proline (Pro), serine (Ser), and threonine (Thr) were the four most prevalent (Figure S2a).” was corrected to “Codons encoding leucine (Leu) was the most prevalent (Figure S2a).”.

“A similar codon usage bias was observed in the RSCU values of most PCGs (Figure S2b).” was corrected to “A similar codon usage bias was observed in the RSCU values of most codon families (Figure S2b).”.

“Among the less frequently used codons, 16 ended in T and 14 ended in G.” was corrected to “Among the less frequently used codons, 16 ended in U and 14 ended in G.”.

Section 3.3, Paragraph 1: Change due to correction of the mitogenome sequence and cleaned datasets.

“The neutrality plot showed that GC3 values ranged from 43.5 to 46.4%, with a slope value of 0.36 (Figure 2a).” was corrected to “The neutrality plot showed that GC3 values ranged from 42.6 to 52.0%, with a slope value of 0.14 (Figure 2a).”.

Section 3.5, Paragraph 1: Change due to correction of the mitogenome sequence and change in the ND3 selection result due to an alignment-related dataset error.

“K_a_/K_s_ analysis showed that *ND3* and *ATP8* had the highest average synonymous substitution rates (K_s_), whereas *ND3* had the lowest non-synonymous substitution rate (K_a_).” was corrected to “K_a_/K_s_ analysis showed that *ATP8* had the highest average non-synonymous substitution rates (K_a_), whereas the average synonymous substitution rates (K_s_) were relatively similar among PCGs.”.

“Consequently, *ND3*’s K_a_/K_s_ ratio was significantly higher than 1 (Figure S5c). By contrast, the K_a_/K_s_ ratios for most other PCGs ranged from 0.017 to 0.220 (Table 1).” was corrected to “Consequently, *ATP8* exhibited the highest K_a_/K_s_ ratio (0.322), while the K_a_/K_s_ ratios of the other PCGs ranged from 0.024 to 0.201 (Table 1 and Figure S5a–c).”.

“Evolutionary distance estimation among the 24 accipitrid species revealed that *ATP8* had the largest K2P genetic distance values, followed by *ND6* and *ND2*, whereas *COI* had the lowest value (Figure S5d).” was corrected to “Evolutionary distance estimation among the 24 accipitrid species revealed that *ATP8* had the largest K2P genetic distance values, followed by *ND3* and *ND6*, whereas *COI* had the lowest value (Figure S5d).”.

Section 3.6, Paragraph 1: Change due to correction of the mitogenome sequence.

“Phylogenetic analyses using 12 concatenated PCGs revealed largely consistent topologies across the accipitrid species for both ML and BI methods (Figures S6 and S7).” was corrected to “Phylogenetic analyses using 12 concatenated PCGs revealed consistent topologies across the accipitrid species for both ML and BI methods (Figures S6 and S7).”.

“A placement difference was exhibited by *Aquila nipalensis*. The ML method placed it with Accipitrinae and Buteoninae, whereas the BI method positioned it closer to *Circaetus pectoralis* and *G. fulvus*.” was corrected to “*Circaetus pectoralis* and *G. fulvus* were clustered together, whereas *Aquila nipalensis* was placed with Accipitrinae and Buteoninae.”.

Section 3.7, Paragraph 1: Change due to reanalysis with the correct dataset for motif assignment.

“The motifs corresponding to the conserved sequences are displayed in CR1 (MEME-03 overlaps with parts of the F-box, D-box, and CSBA; MEME-06 overlaps with parts of the D-box and C-box; MEME-07 corresponds to the bird-box region; and MEME-08 corresponds to the CSBb region).” was corrected to “The motifs corresponding to the conserved sequences are displayed in CR1 (MEME-03 overlaps with parts of the F-box, D-box, and CSBA; MEME-05 overlaps with parts of the D-box and C-box; MEME-06 corresponds to the bird-box region; and MEME-07 corresponds to the CSBb region).”.

### 1.4. Discussion

Section 4, Paragraph 1: Change due to correction of the mitogenome sequence.

“Using 24 mitogenomes, structural variation of mitogenomes and a robust phylogeny derived from PCGs of accipitrid birds were obtained, revealing several phylogenetic relationships that differ from those reported in previous studies [9,47].” was corrected to “Using 24 mitogenomes, structural variation of mitogenomes and a robust phylogeny derived from PCGs of accipitrid birds were obtained, revealing phylogenetic relationships that were consistent with previous studies [43,48,49].”.

“For example, Aquilinae clustered with the clades Accipitrinae and Buteoninae. The current ML analyses supported the previous topologies [42,47]. However, the BI method placed *A. nipalensis*, a representative of Aquilinae, as a sister to the assemblage of *C. pectoralis*, representing Circaetinae, and *G. fulvus*, representing Aegypiinae. This discrepancy may have resulted from other analyses that used fewer sequences and genes, potentially influencing the inferred phylogenies.” was corrected to “The recovered placement of *C. pectoralis*, *G. fulvus*, and *A. nipalensis* supported the previous topologies [43,48].”.

“The mitogenomes of *H. indus* and *A. badius poliopsis* were highly congruent with those of other accipitrids, exhibiting negligible A-skew (ranging from 0.0568 to 0.1532) and significant C-skew (ranging from −0.4239 to −0.3752).” was corrected to “The mitogenomes of *H. indus* and *A. badius poliopsis* were highly congruent with those of other accipitrids, exhibiting negligible A-skew (ranging from 0.0568 to 0.1532) and significant C-skew (ranging from −0.4239 to −0.3642).”.

Section 4.1: Paragraph 1: “Re-examination of the mitogenomes of *P. ptilorhynchus orientalis* (LC541458) and *E. caeruleus* (OK662584) showed that their duplicate CRs had similar sequences within the species, with non-degenerate CR2s. This differed from *P. ptilorhynchus* (MK043029), as described previously [9].” was corrected to “Re-examination of the mitogenomes of *P. ptilorhynchus orientalis* (LC541458.1) and *E. caeruleus* (OK662584.1) showed that their duplicate CRs had similar sequences within the species, with non-degenerate CR2s. This differed from *P. ptilorhynchus* (MK043029.1), as described previously [9].”

Section 4.2: Change due to correction of the mitogenome sequence and change in the ND3 selection result due to an alignment-related dataset error.

Section title

“*4.2. Positive Selection in ND3 and Large Variation in Codon Bias of the ATP8 Gene in Accipitrid Birds*” was corrected to “*4.2. Large Variation in Codon Bias of the ATP8 Gene in Accipitrid Birds*”

Paragraph 1

“In this study, codon families for leucine (Leu), proline (Pro), serine (Ser), and threonine (Thr) were identified as the most prevalent.” was corrected to “In this study, the codon family for leucine (Leu) was identified as the most prevalent.”.

“Most PCGs among the 24 accipitrid mitogenomes showed similar codon usage biases, with the ENc graphs resembling those of other Cathartiformes birds.” was corrected to “The 24 accipitrid mitogenomes showed similar codon usage biases with those of other Cathartiformes and Accipitriformes birds [59].”.

“Translational efficiency indicated a high selection potential of codon bias for *ND2*, *ND3*, *COI*, and *COII* genes (P2 > 5), whereas *ATP8* showed a large P2 variation.” was corrected to “Translational efficiency indicated a high selection potential of codon bias for *ND2*, *ND3*, *COI*, and *COII* genes (P2 > 0.5), whereas *ATP8* showed a large P2 variation.”.

“The neutrality plot showed a GC3 slope of 0.36, indicating 36% mutation pressure and 64% evolutionary selection pressure.” was corrected to “The neutrality plot showed a GC3 slope of 0.14, indicating 14% mutation pressure and 86% evolutionary selection pressure.”.

“By contrast, *ND3*’s K_a_/K_s_ ratio was significantly higher than 1, indicating positive selection. The results suggest that, in accipitrid birds, positive selection drives mitochondrial genes to adapt better to the energy requirements of flight.” was removed.

“However, whether the positive selection in *ND3*, the large variation in codon bias of the *ATP8* gene, and the degeneration of CR2 in accipitrid birds correlate with DNA replication and functional aspects, or are merely coincidental, still remains unclear.” was corrected to “However, whether the large variation in codon bias of the *ATP8* gene and the degeneration of CR2 in accipitrid birds correlate with DNA replication and functional aspects, or are merely coincidental, still remains unclear.”.

“Comparative functional mitogenomics of *ATP8*, *ND3*, and both CRs would be required in future to gain insights into the mitogenome evolution in accipitrid birds.” was corrected to “Comparative functional mitogenomics of *ATP8* and both CRs would be required in future to gain insights into the mitogenome evolution in accipitrid birds.”.

### 1.5. Conclusions

Section 5, Paragraph 1: Change due to correction of the mitogenome sequence and change in the ND3 selection result due to an alignment-related dataset error.

“In addition, positive selection for *ND3* and variable codon usage for *ATP8* indicated adaptive changes in energy metabolism.” was corrected to “In addition, variable codon usage for *ATP8* indicated adaptive changes in energy metabolism.”.

## 2. Modified Main Text Figures

### 2.1. Figure 1

In the original publication, Figure 1a was generated based on the incorrect *H. indus* mitogenome sequence. After reanalysis, the updated figure has been reconstructed using the corrected dataset. The corrected Figure 1 appears below.

### 2.2. Figure 2

In the original publication, Figure 2a–c were analyzed using datasets containing an incorrect *H. indus* mitogenome sequence. After reanalysis, the figures have been updated. The corrected Figure 2 appears below.

### 2.3. Figure 3

In the original publication, Figure 3 was analyzed using datasets containing an incorrect *H. indus* mitogenome sequence. After reanalysis, the figure has been updated. The corrected Figure 3 appears below.

### 2.4. Figure 4

In the original publication, Figure 4 was analyzed using datasets containing an incorrect *H. indus* mitogenome sequence. After reanalysis, the figure has been updated. The corrected Figure 4 appears below.

## 3. Modified Main Text Tables

### 3.1. Table 1

In the original publication, Table 1 was analyzed using datasets containing an incorrect *H. indus* mitogenome sequence. The columns ‘Gene’ and ‘Substitution Model’ were sorted alphabetically by gene name, while the other columns were ordered according to the mitochondrial OXPHOS complexes. After reanalysis with the corrected sequence and appropriate setting, Table 1 has been updated and consistently ordered according to the mitochondrial OXPHOS complexes.

### 3.2. Table 2

In the original publication, the entries for *H. indus* in Table 2 were analyzed using an incorrect *H. indus* mitogenome sequence. After reanalysis with the corrected sequence, the pairwise identity value for this species has been updated. The corrected Table 2 appears below.

## 4. Modified Supplementary Materials

The Supplementary Figures S1–S9 and Tables S1–S3 have been updated. A new supplementary file entitled “Detailed methods for the mitogenome sequencing and assembly of *H. indus*.”has been added.

## 5. Missing Citation

### 5.1. Materials and Methods, Section 2.2

Jin et al. (2020) has been inserted as [19] in Paragraph 1.

“For *H. indus*, Illumina resequencing was performed twice, and the combined raw reads were used for de novo assembly of a mitochondrial backbone with GetOrganelle v1.7.4.1 (dependencies: Bowtie2, SPAdes, BLAST) [19].”

### 5.2. Materials and Methods, Section 2.5

Paradis (2010) has been inserted as [35] and Tamura et al. (2021) as [34] in Paragraph 1.

“The mean genetic distance between the annotated PCGs of the studied mitogenomes was calculated using the Kimura-2-parameter (K2P) substitution model in MEGA 11 [34], and nucleotide diversity was estimated using the R package “pegas” [35].”

### 5.3. Materials and Methods, Section 2.6

Rambaut et al. (2018) has been inserted as [45] in Paragraph 1.

“Markov chain Monte Carlo simulations were run for 10 million iterations, and the convergence was verified using Tracer v1.7.2 [45].”

### 5.4. Discussion, Section 4.2

De Panis et al. (2021) has been inserted as [59] in Paragraph 1.

“The 24 accipitrid mitogenomes showed similar codon usage biases with those of other Cathartiformes and Accipitriformes birds [59].”

**References**

19 Jin, J.-J.; Yu, W.-B.; Yang, J.-B.; Song, Y.; dePamphilis, C.W.; Yi, T.-S.; Li, D.-Z. GetOrganelle: A fast and versatile toolkit for accurate de novo assembly of organelle genomes. *Genome Biol.* **2020**, *21*, 241. https://doi.org/10.1186/s13059-020-02154-5.
34 Tamura, K.; Stecher, G.; Kumar, S. MEGA11: Molecular Evolutionary Genetics Analysis Version 11. *Mol. Biol. Evol.* **2021**, *38*, 3022–3027. https://doi.org/10.1093/molbev/msab120.
35 Paradis, E. pegas: An R Package for Population Genetics with an Integrated-Modular Approach. *Bioinformatics* **2010**, *26*, 419–420. https://doi.org/10.1093/bioinformatics/btp696.
45 Rambaut, A.; Drummond, A.J.; Xie, D.; Baele, G.; Suchard, M.A. Posterior Summarization in Bayesian Phylogenetics Using Tracer 1.7. *Syst. Biol.* **2018**, *67*, 901–904. https://doi.org/10.1093/sysbio/syy032.
59 De Panis, D.; Lambertucci, S.A.; Wiemeyer, G.; Speziale, K.L.; Damico, N.L.; Johnson, J.A.; Mindlin, G.B.; Rebolo-Ifrán, N. Mitogenomic Analysis of Extant Condor Species Provides Insight into the Molecular Evolution of Vultures. *Sci. Rep.* **2021**, *11*, 17109. https://doi.org/10.1038/s41598-021-96080-6.

With this correction, the order of some references has been adjusted accordingly. 

## 6. Data Availability Statement Updated

The corrected mitochondrial genome has been deposited in GenBank under accession number OP133375.2.

## 7. Affiliation of Author Updated

The affiliation (No. 6) of Aingorn Chaiyes has been updated to “The International Undergraduate Program in Bioscience and Technology, Faculty of Science, Kasetsart University, Bangkok 10900, Thailand”.

## Figures and Tables

**Figure 1:**
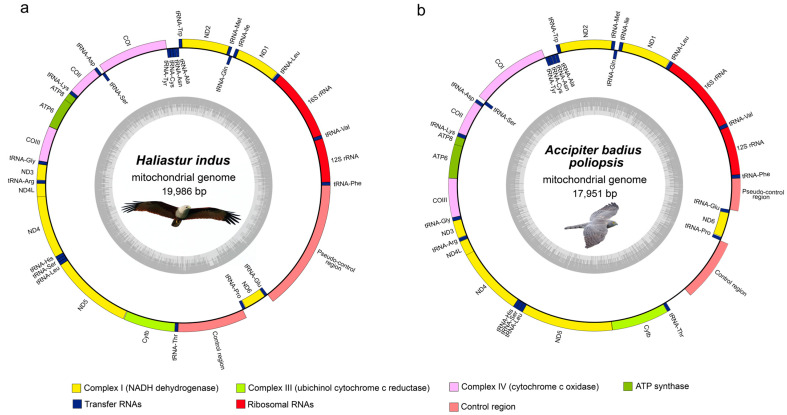
Circular maps of (**a**) *H. indus* and (**b**) *A. badius poliopsis* mitochondrial genomes.

**Figure 2:**
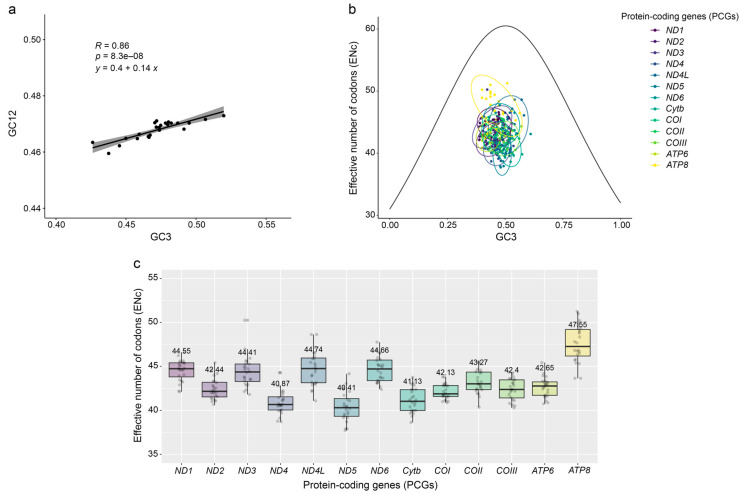
Codon analysis results of 13 protein-coding genes (PCGs) from 24 species within the Accipitridae family: (**a**) neutrality plot; (**b**) effective number of codons (ENc) plot; and (**c**) box plot of ENc of 24 species in each PCGs.

**Figure 3:**
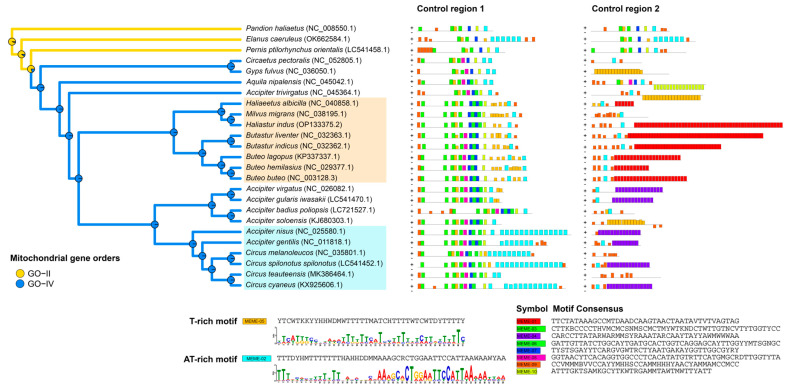
Mitochondrial gene order and collections of DNA sequence motifs found in both CR1 and CR2 regions. Mitochondrial gene orders (GO-II and GO-IV) mapped onto a phylogenetic tree based on 12 protein-coding genes (PCGs) are shown on the left, whereas the distribution of conserved motifs within control region 1 (CR1) and control region 2 (CR2/pseudo-CR) is shown on the right. Colored boxes indicate conserved motifs identified by MEME. Plus (+) and minus (−) symbols denote forward and reverse strand orientations, respectively. Lineages associated with the T-rich (MEME-05) and AT-rich (MEME-02) motifs are highlighted in orange and light blue boxes on the phylogenetic tree.

**Figure 4:**
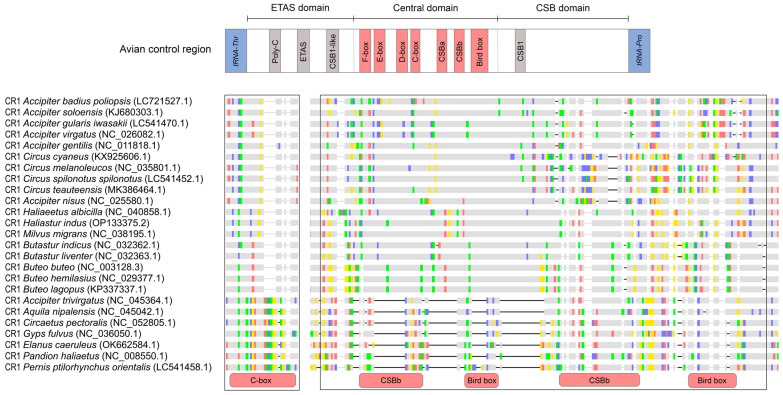
Nucleotide sequence alignment of the conserved sequence element in CR1. The upper schematic represents the avian control region, with conserved sequence boxes (F, E, D, C, and bird), CSBa, and CSBb in the central domain, shown in red. Conserved sequence elements in other domains are shown in grey. The boxed region highlights sequence variations with corresponding annotations. Colored blocks represent nucleotide differences (A, C, G, and T), whereas grey blocks indicate identical nucleotides.

**Table 1:** Genetic distance and non-synonymous/synonymous (K_a_/K_s_) values for 13 PCGs across 24 accipitrid mitogenomes.

Gene	Substitution Model	K2P Genetic Distance	Nucleotide Diversity (π)	K_a_	K_s_	K_a_/K_s_ Ratio
*ND1*	HKY + G + I	0.15964	0.14064	0.03637	0.52465	0.06933
*ND2*	TN93 + G + I	0.17310	0.15034	0.07250	0.45150	0.16057
*ND3*	HKY + G	0.19368	0.15984	0.09606	0.47800	0.20095
*ND4*	HKY + G + I	0.15160	0.13486	0.04622	0.44860	0.10304
*ND4L*	HKY + G	0.16407	0.14272	0.05371	0.47831	0.11229
*ND5*	GTR + G + I	0.15378	0.13623	0.05898	0.44092	0.13376
*ND6*	HKY + G + I	0.18927	0.16228	0.06739	0.56474	0.11932
*COI*	GTR + G + I	0.12199	0.10999	0.01112	0.46565	0.02389
*COII*	TN93 + G + I	0.12287	0.10987	0.02295	0.43864	0.05233
*COIII*	TN93 + G + I	0.12388	0.11193	0.02549	0.43445	0.05868
*Cytb*	GTR + G + I	0.13520	0.12148	0.02928	0.46563	0.06288
*ATP6*	HKY + G + I	0.15753	0.13885	0.05554	0.43703	0.12708
*ATP8*	TN93 + G + I	0.24230	0.19855	0.15808	0.49053	0.32226

**Table 2:** The structure of control regions in the mitogenome of raptors in the Accipitridae and Pandionidae families.

Lineages	Species	CR1			CR2/Pseudo-CR			Pairwise Identity (%)
		ETAS	Central	CSB	ETAS	Central	CSB	
Family Accipitridae								
Circinae	*Circus cyaneus*	🗸	🗸	🗸	-	-	-	18.5
	*Circus teauteensis*	🗸	🗸	🗸	-	-	-	27.2
	*Circus spilonotus spilonotus*	🗸	🗸	🗸	-	-	-	17.5
	*Circus melanoleucos*	🗸	🗸	🗸	-	-	-	12.0
Accipitrinae	*Accipiter gentilis*	🗸	🗸	🗸	-	-	-	18.4
	*Accipiter nisus*	🗸	🗸	🗸	-	-	-	18.7
	*Accipiter soloensis*	🗸	🗸	🗸	-	-	-	29.5
	*A. badius poliopsis*	🗸	🗸	🗸	-	-	-	15.8
	*Accipiter gularis iwasakii*	🗸	🗸	🗸	-	-	-	33.5
	*Accipiter virgatus*	🗸	🗸	🗸	-	-	-	32.1
Buteonninae	*Buteo buteo*	🗸	🗸	🗸	-	-	-	36.5
	*Buteo hemilasius*	🗸	🗸	🗸	-	-	-	14.1
	*Buteo lagopus*	🗸	🗸	🗸	-	-	-	28.5
	*B. indicus*	🗸	🗸	🗸	-	-	-	17.4
	*Butastur liventer*	🗸	🗸	🗸	-	-	-	15.9
	*M. migrans*	🗸	🗸	🗸	-	-	-	23.0
	*H. indus*	🗸	🗸	🗸	-	-	-	38.5
	*Haliaeetus albicilla*	🗸	🗸	🗸	-	-	-	24.3
Accipitrinae	*Accipiter trivirgatus*	🗸	🗸	🗸	-	-	-	27.4
Aquilinae	*A. nipalensis*	🗸	🗸	🗸	-	-	-	36.5
Aegypiinae	*G. fulvus*	🗸	🗸	🗸	-	-	-	30.0
Circaetinae	*C. pectoralis*	🗸	🗸	🗸	-	-	-	24.6
Perninae	*P. ptilorhynchus orientalis*	🗸	🗸	🗸	🗸	🗸	🗸	88.9
Elaninae	*E. caeruleus*	🗸	🗸	🗸	🗸	🗸	🗸	78.4
Family Pandionidae								
	*P. haliaetus*	🗸	🗸	🗸	🗸	🗸	🗸	87.3
