# Supplementary material for: Insights into Mitochondrial Rearrangements and Selection in Accipitrid Mitogenomes, with New Data on Haliastur indus and Accipiter badius poliopsis
Source: Genes (Basel). 2024 Nov 7;15(11):1439. doi: 10.3390/genes15111439 (PMC11593783; doi:10.3390/genes15111439)
Supplement: Supplementary file 1 [file genes-15-01439-s001.zip › genes-3275452-Supplementary Material-Detailed methods for the mitogenome sequencing and assembly of H. indus.pdf]

Mitogenome Sequencing and Assembly of *Haliastur indus*

Whole-genome resequencing of the same sample was performed using 100 ng/μL DNA on the Illumina NovaSeq™ 6000 platform (2 × 150 bp paired-end, 15× coverage) at Novogene Co., Ltd. (Singapore). Raw reads from this run were combined with previous data to increase coverage for assembly. De novo assembl was generated with GetOrganelle v1.7.4.1 (Jin et al., 2020) (dependencies: Bowtie2 v2.3.5.1, SPAdes v3.15.4, BLAST v2.9.0).

Control region fragments were amplified using primers (Figure S10, Table S4): (i) the terminal portion of the first control region and (ii) the second control region, which was subdivided into 5′ and 3′ fragments. PCR reactions for the partial CR1 fragment (1,189 bp) were performed in 15 μL volumes containing 1× Ex Taq Buffer (Mg^{2+} plus, 2 mM), 0.2 mM dNTPs, 0.3 μM primers, 0.75 U TaKaRa Ex Taq® DNA Polymerase (Takara Bio Inc., Shiga, Japan), and 25 ng genomic DNA. The partial CR2 fragments (713 bp, 5′ region; ≥1098 bp, 3′ region) were amplified in 50 μL volumes containing 1× KOD FX Neo buffer, 0.4 mM dNTPs, 0.3 μM primers, 1.0 U KOD FX Neo (Toyobo Co., Ltd., Osaka, Japan), and 25 ng genomic DNA. PCR cycling conditions were as follows: initial denaturation at 94 °C for 5 min; 35 cycles of 94 °C for 30 s, annealing (Ta) for 30 s, and 72 °C for 30 s; with a final extension at 72 °C for 5 min. To reduce non-specific amplification, touchdown PCR was applied for the partial CR2 3′ fragment, with annealing temperatures reduced from 68 °C to 60 °C over the first 8 cycles, followed by 25 cycles at a stable 60 °C.

The partial CR1 fragment and the CR2 5′ fragment were sequenced using an ABI 3730XL automatic sequencer (Sanger sequencing), while the CR2 3′ fragment was sequenced using PromethION P2i (Oxford Nanopore Technologies, ONT, Oxford, UK). Nanopore reads were processed using Fastplong v.0.2.2 (Chen, 2023) with the options -3 -5 -x --length_required 2500 --length_limit 3500, which stands for 3' and 5' quality trimming, polyX trimming, and filtering only reads between 2500 and 3500 bp long. The processed reads were ussembled using canu v.2.3 (Koren et al., 2017) with options genomeSize=3.1k minInputCoverage=2000, which stands for estimated genome size 3100 bp and desired coverage of 2000× and polished with Illumina MiSeq data (2 × 150 bp paired-end, 100× coverage). Finally, all fragments (Illumina, Sanger, and Nanopore) were merged into the final assembly using Geneious Assembler v2025.0.3.

Gene annotation of the *H. indus* mitogenome was performed using MITOS2 (Donath et al., 2019) (Galaxy tool "MITOS2", version 2.0.1+galaxy1) with the vertebrate mitochondrial genetic code (code 2). The analysis was run against the RefSeq63m reference dataset, with annotation of protein-coding genes (PCGs), rRNAs, and tRNAs. Output files were generated in GFF/BED format and subsequently subjected to manual curation to confirm gene boundaries and completeness.

References

Chen, S. (2023). Ultrafast one-pass FASTQ data preprocessing, quality control, and deduplication using fastp. iMeta, 2(2), e107. https://doi.org/10.1002/imt2.107

Donath, A., Jühling, F., Al-Arab, M., Bernhart, S. H., Reinhardt, F., Stadler, P. F., Middendorf, M., & Bernt, M. (2019). Improved annotation of protein-coding genes boundaries in metazoan mitochondrial genomes. Nucleic Acids Research, 47(20), 10543–10552. https://doi.org/10.1093/nar/gkz833

Jin, J.-J., Yu, W.-B., Yang, J.-B., Song, Y., dePamphilis, C. W., Yi, T.-S., & Li, D.-Z. (2020). GetOrganelle: a fast and versatile toolkit for accurate de novo assembly of organelle genomes. Genome Biology, 21(1), 241. https://doi.org/10.1186/s13059-020-02154-5

Koren, S., Walenz, B. P., Berlin, K., Miller, J. R., Bergman, N. H., & Phillippy, A. M. (2017). Canu: scalable and accurate long-read assembly via adaptive k-mer weighting and repeat separation. Genome Research, 27(5), 722–736. https://doi.org/10.1101/gr.215087.116

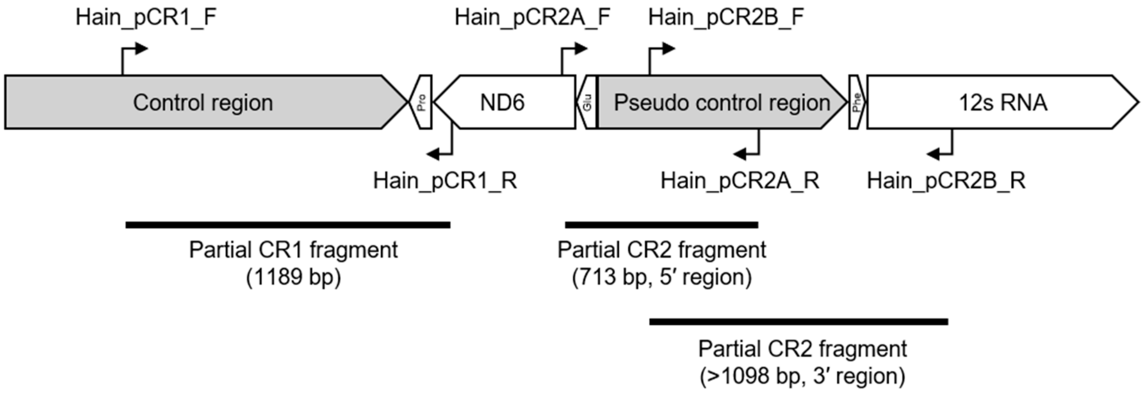

Figure S10. Schematic illustration of a portion of the *Haliastur indus* mitogenome showing the positions of primers (arrows) used in this study.

Table S4. List of primers used for PCR amplification

Primer name	Sequence (5′ – 3′)	Ta (˚C)	Expected size
Hain_pCR1_F	TCCATTCCCAGCTTCAGGACCA	59	1,189 bp
Hain_pCR1_R	TGGGGGTTGCTGTTGACGTT		
Hain_pCR2A_F	AGCCCCCTAAAACAAACCCCACTC	57	713 bp
Hain_pCR2A_R	TGGTTGATGCATTTGGTAGGGGAGT		
Hain_pCR2B_F	AAGGCAGAATCCCCGCACACAA	60	At least 1,098 bp
Hain_pCR2B_R	TGCCCGCTCTTTACGCCGAATA		
