# Supplementary material for: Insights into Mitochondrial Rearrangements and Selection in Accipitrid Mitogenomes, with New Data on Haliastur indus and Accipiter badius poliopsis
Source: Genes (Basel). 2024 Nov 7;15(11):1439. doi: 10.3390/genes15111439 (PMC11593783; doi:10.3390/genes15111439)
Supplement: Supplementary file 1 [file genes-15-01439-s001.zip › genes-3275452-updated Supplementary Figures S1-S9.pdf]

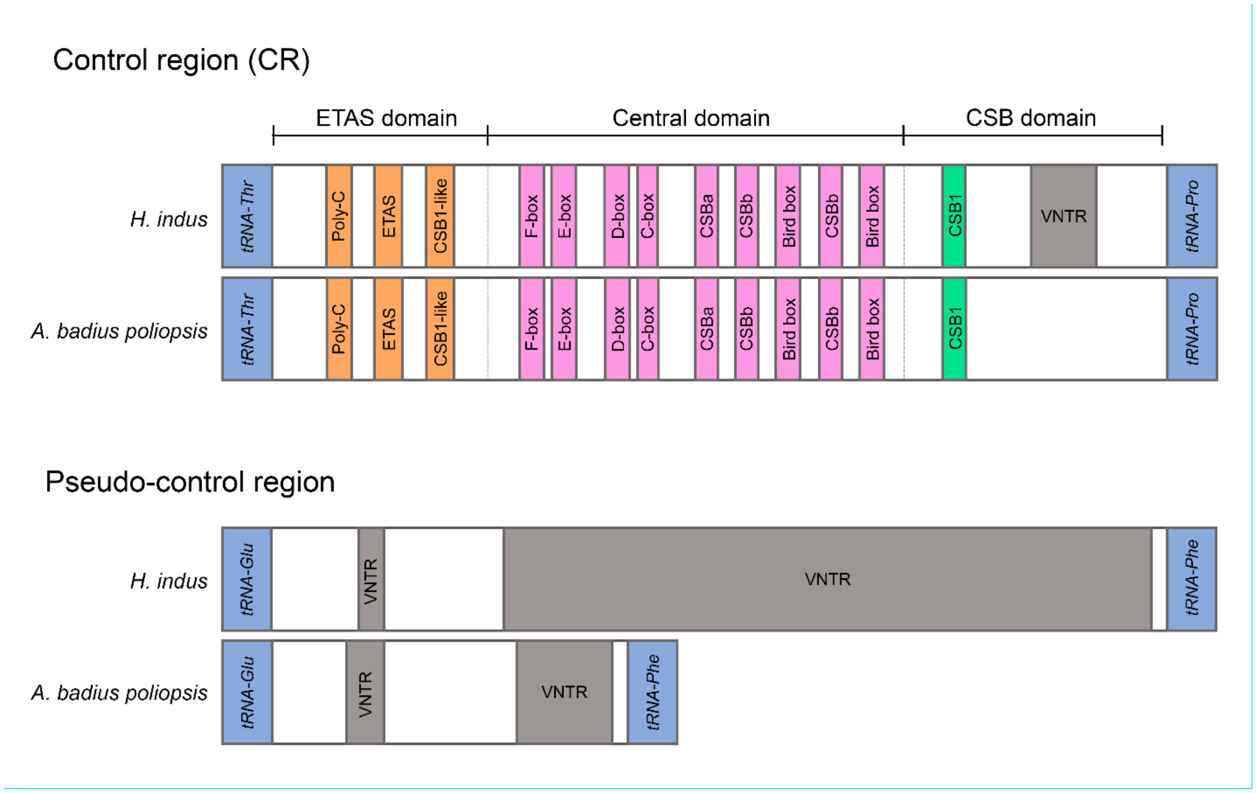

Figure S1. Structure of mitochondrial control region of *Haliastur indus* and *Accipiter badius poliopsis*

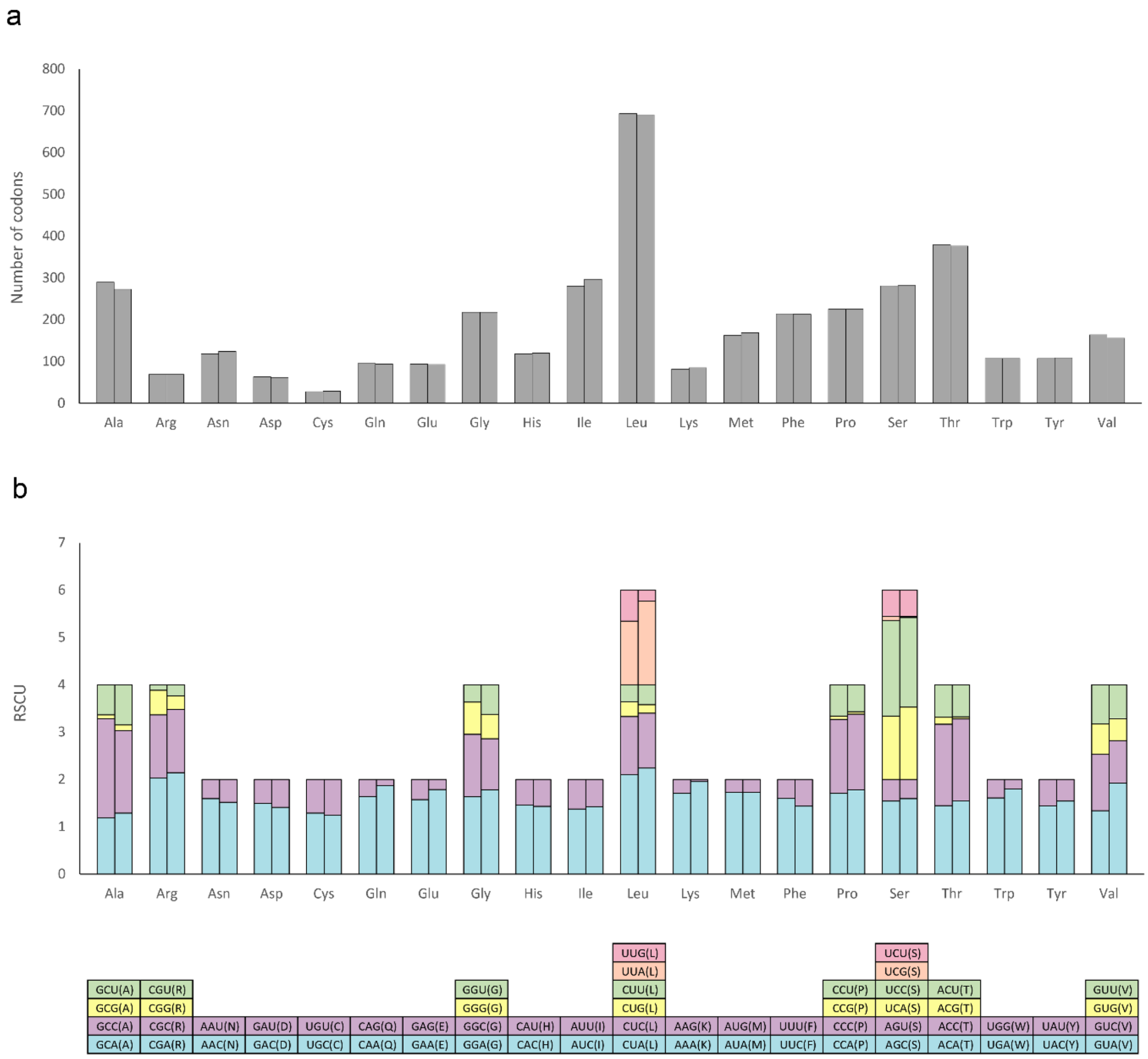

Figure S2. Codon usage and Relative Synonymous Codon Usage (RSCU) in *Haliastur indus* and *Accipiter badius poliopsis*

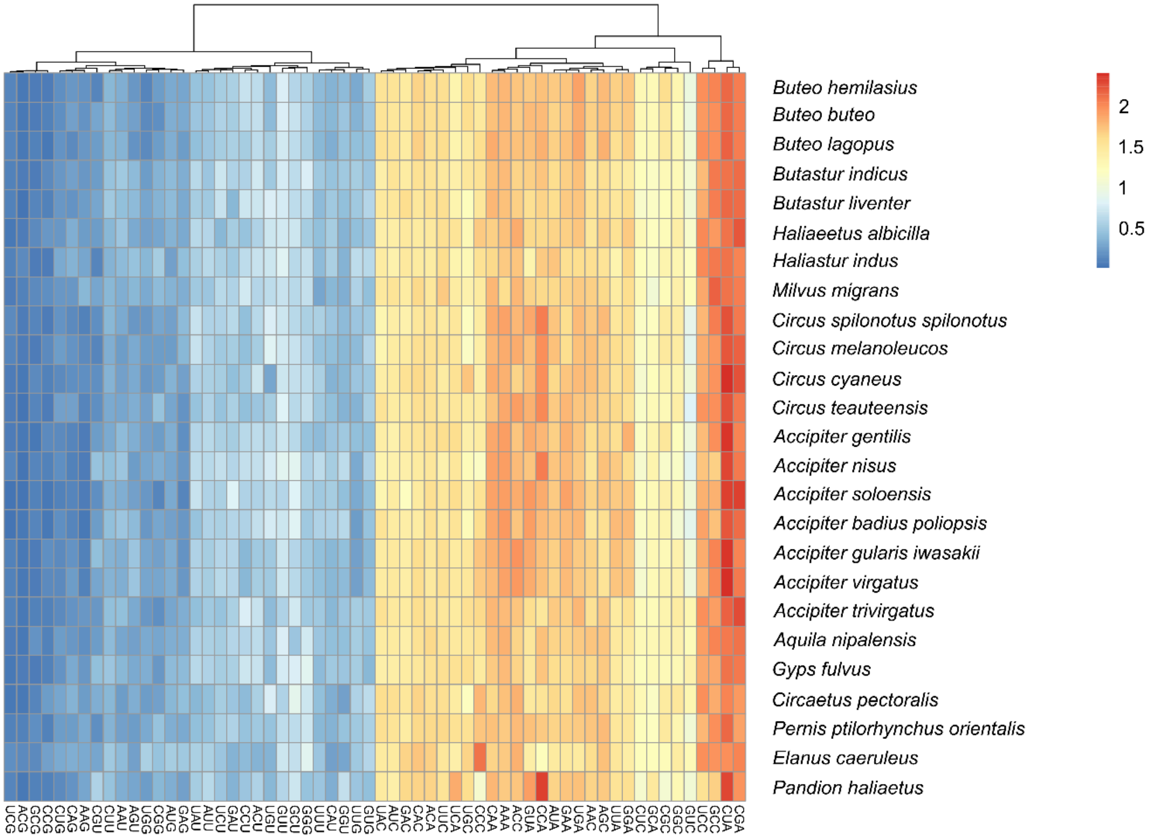

Figure S3. Heat map of Relative Synonymous Codon Usage of raptor species in the family Accipitridae.

a

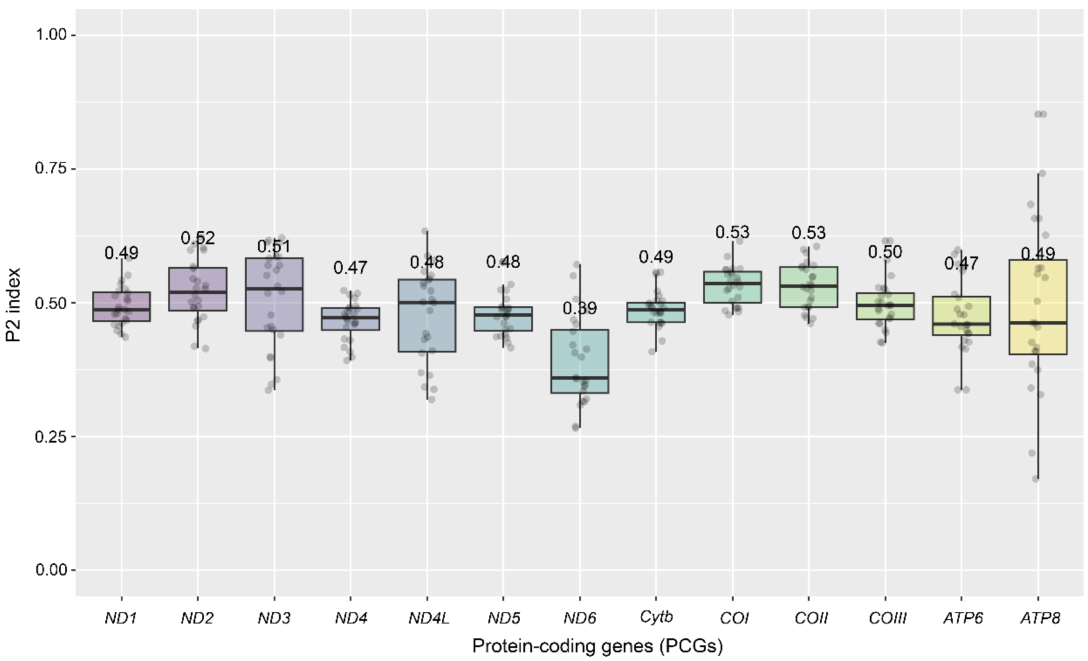

b

Subfamily	Species	*ND1*	*ND2*	*ND3*	*ND4*	*ND4L*	*ND5*	*ND6*	*Cytb*	*COI*	*COII*	*COIII*	*ATP6*	*ATP8*
Buteoninae	*Buteo hemilasius*	0.50	0.53	0.61	0.49	0.34	0.46	0.35	0.52	0.54	0.49	0.52	0.56	0.45
	Buteo buteo	0.52	0.54	0.62	0.52	0.44	0.49	0.41	0.51	0.53	0.49	0.53	0.57	0.41
	Buteo lagopus	0.47	0.51	0.59	0.49	0.34	0.49	0.31	0.56	0.53	0.49	0.49	0.57	0.41
	Butastur indicus	0.54	0.42	0.56	0.45	0.55	0.53	0.33	0.46	0.48	0.56	0.50	0.44	0.46
	Butastur liventer	0.49	0.46	0.58	0.47	0.41	0.58	0.27	0.49	0.55	0.61	0.50	0.45	0.42
	Haliaeetus albicilla	0.46	0.50	0.48	0.40	0.37	0.52	0.35	0.43	0.49	0.55	0.50	0.48	0.34
	Haliastur indus	0.49	0.49	0.52	0.42	0.51	0.44	0.35	0.49	0.54	0.48	0.53	0.49	0.46
	Milvus migrans	0.58	0.53	0.57	0.43	0.54	0.49	0.49	0.48	0.56	0.53	0.48	0.52	0.57
Circinae	*Circus spilonotus spilonotus*	0.47	0.57	0.55	0.39	0.56	0.42	0.42	0.46	0.51	0.53	0.46	0.43	0.17
	Circus melanoleucos	0.48	0.60	0.57	0.41	0.53	0.44	0.36	0.46	0.55	0.46	0.50	0.42	0.22
	Circus cyaneus	0.48	0.47	0.40	0.48	0.44	0.45	0.34	0.50	0.56	0.58	0.53	0.44	0.43
	Circus teauteensis	0.46	0.41	0.44	0.46	0.59	0.47	0.36	0.55	0.56	0.55	0.44	0.49	0.39
Accipitrinae	*Accipiter gentilis*	0.45	0.50	0.45	0.51	0.41	0.52	0.41	0.46	0.50	0.47	0.51	0.46	0.37
	Accipiter nisus	0.48	0.56	0.52	0.52	0.58	0.49	0.51	0.48	0.52	0.57	0.47	0.60	0.55
	Accipiter soloensis	0.49	0.49	0.53	0.43	0.49	0.48	0.32	0.41	0.55	0.53	0.49	0.59	0.63
	Accipiter badius poliopsis	0.53	0.62	0.62	0.46	0.50	0.51	0.55	0.50	0.53	0.60	0.43	0.43	0.50
	Accipiter gularis iwasakii	0.55	0.60	0.36	0.48	0.54	0.47	0.31	0.49	0.49	0.57	0.45	0.34	0.66
	Accipiter virgatus	0.54	0.60	0.34	0.48	0.54	0.47	0.31	0.48	0.49	0.57	0.43	0.34	0.66
	Accipiter trivirgatus	0.51	0.53	0.59	0.47	0.63	0.45	0.40	0.50	0.49	0.59	0.58	0.41	0.85
Aquilinae	*Aquila nipalensis*	0.46	0.49	0.45	0.49	0.36	0.43	0.27	0.50	0.56	0.50	0.55	0.48	0.74
Aegypiinae	*Gyps fulvus*	0.52	0.47	0.35	0.47	0.52	0.49	0.57	0.48	0.59	0.53	0.62	0.46	0.56
Circaetinae	*Circaetus pectoralis*	0.51	0.54	0.46	0.49	0.50	0.49	0.45	0.46	0.56	0.52	0.47	0.51	0.55
Perninae	*Pernis ptilorhynchus orientalis*	0.44	0.61	0.61	0.46	0.43	0.44	0.47	0.48	0.62	0.51	0.50	0.46	0.68
Elaninae	*Elanus caeruleus*	0.44	0.47	0.40	0.51	0.32	0.43	0.46	0.50	0.48	0.47	0.46	0.46	0.33

Min 0.50 Max

Figure S4. Translational efficiency of 13 protein-coding genes (PCGs) among 24 species within the Accipitridae family

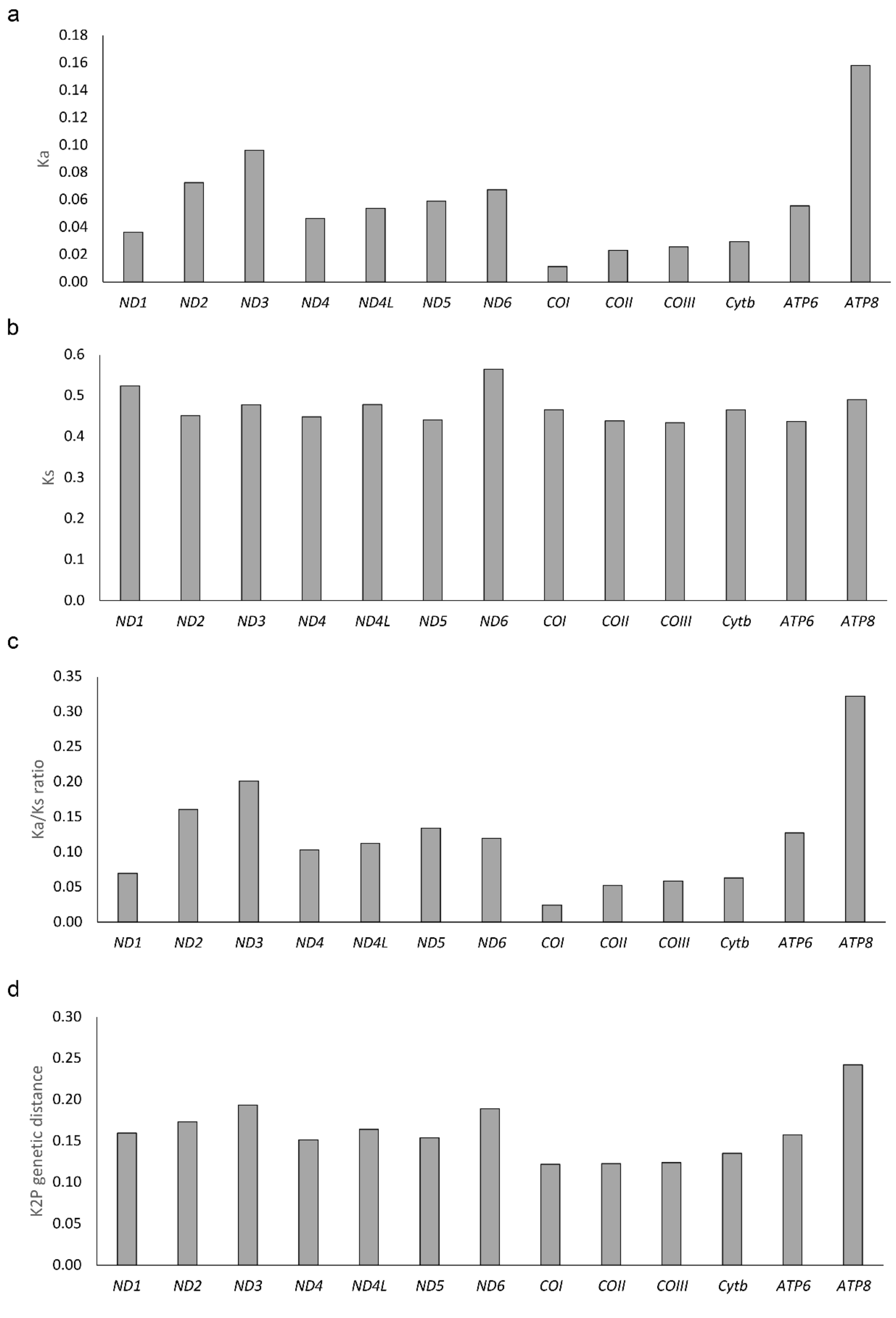

Figure S5. K2P genetic distance and the Ka/Ks ratio of 13 PCGs among 24 species within the Accipitridae family

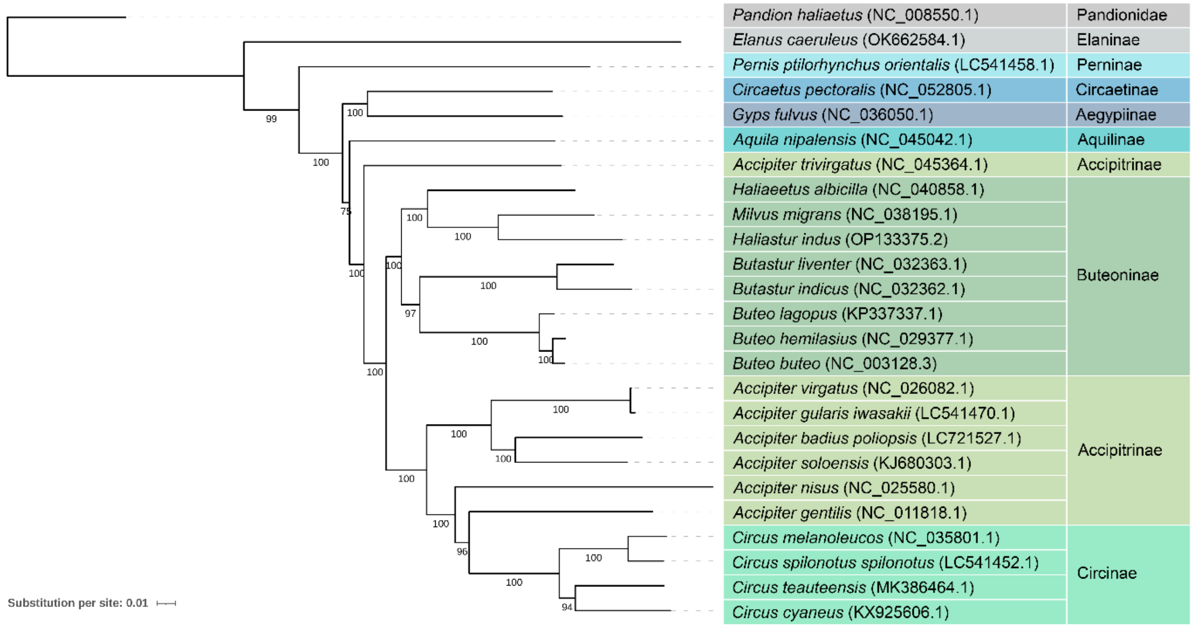

Figure S6. Phylogenetic relationships within the Accipitridae family based on maximum likelihood

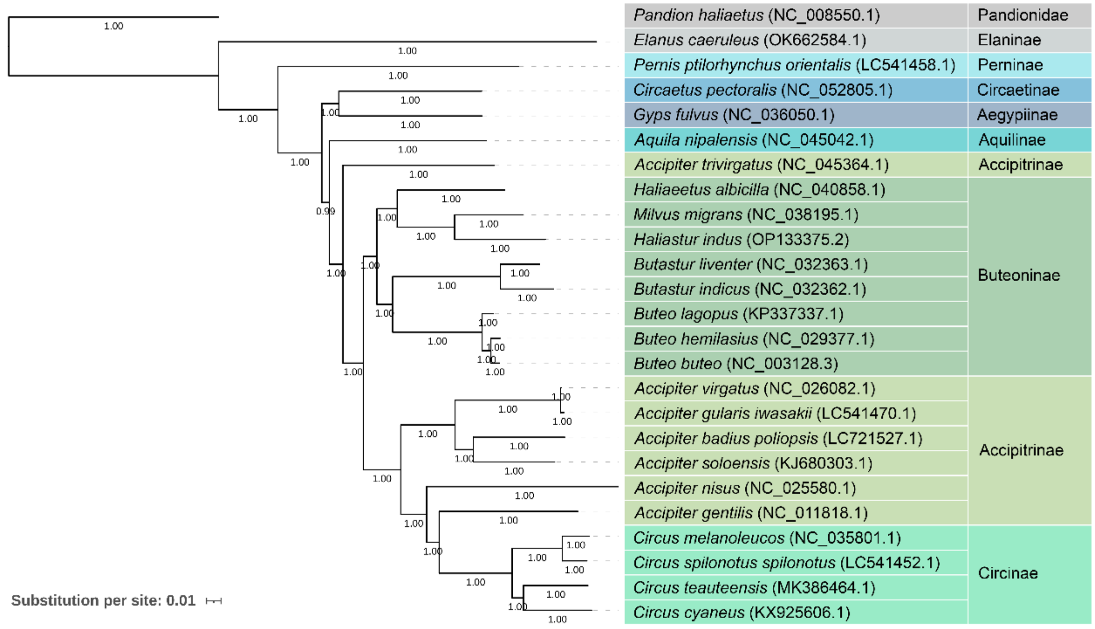

Figure S7. Phylogenetic relationships within the Accipitridae family based on Bayesian inference

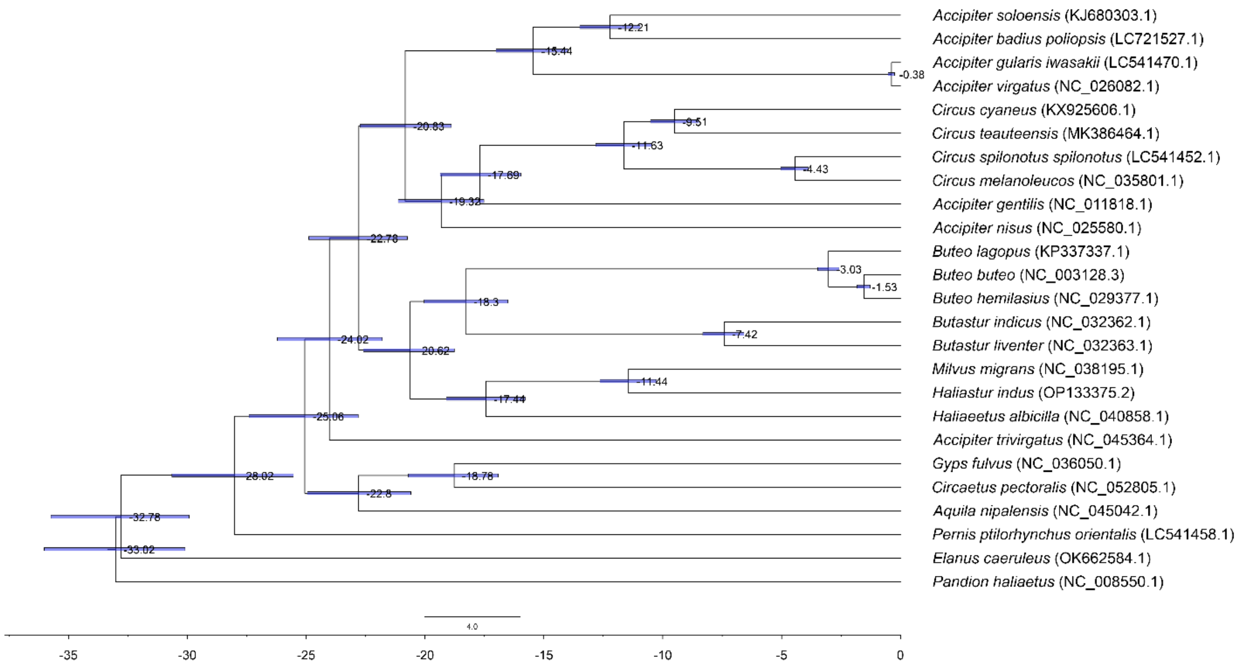

Figure S8. Divergence time estimates within the Accipitridae family

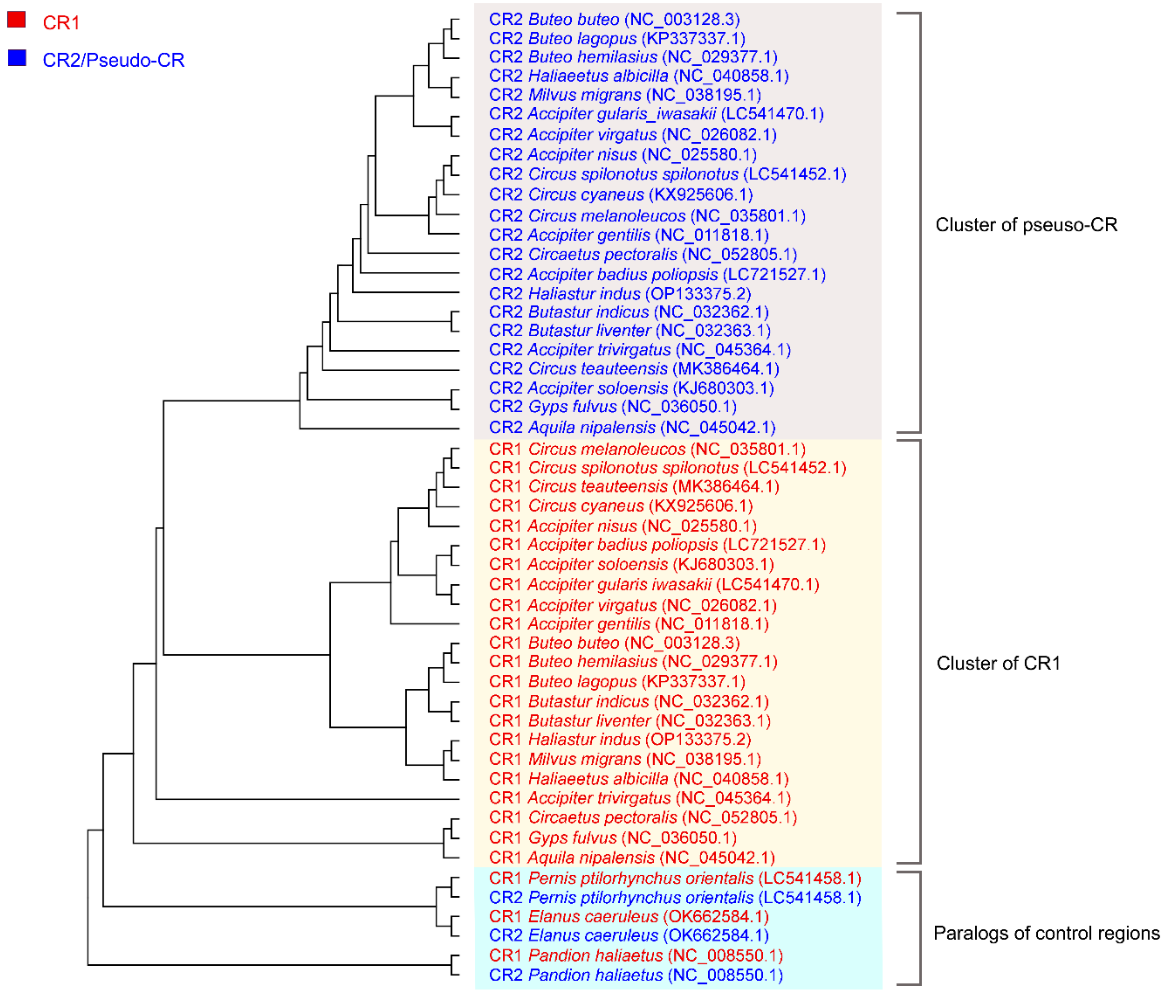

Figure S9. Phylogenetic tree of CR1 and CR2 inferred by neighbor-joining method
