# Supplementary material for: Insights into Mitochondrial Rearrangements and Selection in Accipitrid Mitogenomes, with New Data on Haliastur indus and Accipiter badius poliopsis
Source: Genes (Basel). 2024 Nov 7;15(11):1439. doi: 10.3390/genes15111439 (PMC11593783; doi:10.3390/genes15111439)
Supplement: Supplementary file 1 [file genes-15-01439-s001.zip › genes-3275452-updated Supplementary Tables S1-S3.pdf]

Table S1. Nucleotide composition, GC frequency, and skewness of mitogenomes in the family Accipitridae

No.	Species	GenBank accession no.	Size (bp)	Nucleotide composition (%)				AT all (%)	GC all (%)	AT skew	GC skew
				A	T	G	C				
1	*Buteo hemilasius*	NC_029377.1	18,079	30.7	24.2	13.2	32.0	54.9	45.2	0.1184	-0.4148
2	*Buteo buteo*	NC_003128.3	18,674	31.1	24.1	13.2	31.7	55.2	44.9	0.1279	-0.4135
3	*Buteo lagopus*	KP337337.1	18,559	31.1	23.9	13.1	31.9	55.0	45.0	0.1315	-0.4167
4	*Butastur indicus*	NC_032362.1	19,059	30.7	25.1	13.3	30.9	55.8	44.2	0.1012	-0.3966
5	*Butastur liventer*	NC_032363.1	19,673	31.2	25.2	13.6	30.0	56.4	43.6	0.1067	-0.3752
6	*Haliaeetus albicilla*	NC_040858.1	17,719	30.1	24.2	13.8	31.9	54.3	45.7	0.1071	-0.3957
7	*Haliastur indus*	OP133375.2	19,986	30.6	24.5	14.3	30.6	55.1	44.9	0.1107	-0.3642
8	*Milvus migrans*	NC_038195.1	18,016	30.1	24.1	13.6	32.3	54.2	45.9	0.1107	-0.4070
9	*Circus spilonotus spilonotus*	LC541452.1	18,738	31.6	24.4	12.8	31.2	56.0	44.0	0.1278	-0.4178
10	*Circus melanoleucos*	NC_035801.1	17,749	30.9	24.0	13.3	31.8	54.9	45.1	0.1273	-0.4089
11	*Circus cyaneus*	KX925606.1	18,754	31.7	24.1	13.0	31.3	55.8	44.3	0.1362	-0.4136
12	*Circus teauteensis*	MK386464.1	17,873	31.0	22.7	13.6	32.7	53.7	46.3	0.1532	-0.4113
13	*Accipiter gentilis* (*Astur gentilis*)	NC_011818.1	18,266	31.8	24.1	12.7	31.4	55.9	44.1	0.1388	-0.4239
14	*Accipiter nisus*	NC_025580.1	18,647	31.3	25.6	12.8	30.3	56.9	43.1	0.1004	-0.4064
15	*Accipiter soloensis* (*Tachyspiza soloensis*)	KJ680303.1	17,900	29.9	25.9	12.9	31.4	55.8	44.3	0.0708	-0.4180
16	*Accipiter badius poliopsis* (*Tachyspiza badius poliopsis*)	LC721527.1	17,951	30.7	25.0	13.0	31.3	55.7	44.3	0.1032	-0.4139
17	*Accipiter gularis iwasakii* (*Tachyspiza gularis iwasakii*)	LC541470.1	17,784	31.0	23.9	13.5	31.7	54.9	45.2	0.1295	-0.4033
18	*Accipiter virgatus* (*Tachyspiza virgatus*)	NC_026082.1	17,952	31.1	23.9	13.5	31.6	55.0	45.1	0.1301	-0.4010
19	*Accipiter trivirgatus* (*Lophospiza trivirgatus*)	NC_045364.1	18,454	31.2	24.4	13.4	31.0	55.6	44.4	0.1228	-0.3950
20	*Aquila nipalensis*	NC_045042.1	18,450	30.3	23.8	14.0	32.0	54.1	46.0	0.1198	-0.3925
21	*Gyps fulvus*	NC_036050.1	18,094	29.0	25.9	13.8	31.4	54.9	45.2	0.0568	-0.3898
22	*Circaetus pectoralis*	NC_052805.1	17,473	29.6	23.4	14.3	32.7	53.0	47.0	0.1162	-0.3910
23	*Pernis ptilorhynchus orientalis*	LC541458.1	18,340	29.8	23.7	13.8	32.8	53.5	46.6	0.1142	-0.4060
24	*Elanus caeruleus*	OK662584.1	18,898	29.0	24.7	14.3	32.0	53.7	46.3	0.0811	-0.3822

Table S2. Mitochondrial genome organization of *Haliastur indus* and *Tachyspiza badius poliopsis* (*Accipiter badius poliopsis*)

Feature	Strand	Brahminy kite (*Haliastur indus*)						Shikra (*Tachyspiza badius poliopsis*)					
		Position		Length	Start	Stop	Anticodon	Position		Length	Start	Stop	Anticodon
		From	to	(bp)	codons	codons		From	to	(bp)	codons	codons	
tRNA-Phe	H	1	70	70	–	–	GAA	144	214	71	–	–	GAA
12S rRNA	H	71	1,041	971	–	–	–	214	1,189	976	–	–	–
tRNA-Val	H	1,042	1,113	72	–	–	TAC	1,189	1,259	71	–	–	TAC
16S rRNA	H	1,114	2,705	1,592	–	–	–	1,260	2,860	1,601	–	–	–
tRNA-Leu	H	2,706	2,779	74	–	–	TAA	2,860	2,933	74	–	–	TAA
ND1	H	2,789	3,766	978	ATG	AGG	–	2,943	3,920	978	ATG	AGG	–
tRNA-Ile	H	3,765	3,836	72	–	–	GAT	3,919	3,990	72	–	–	GAT
tRNA-Gln	L	3,851	3,921	71	–	–	TTG	4,006	4,076	71	–	–	TTG
tRNA-Met	H	3,921	3,989	69	–	–	CAT	4,076	4,144	69	–	–	CAT
ND2	H	3,990	5,030	1,041	ATG	TAG	–	4,145	5,185	1,041	ATG	TAG	–
tRNA-Trp	H	5,029	5,101	73	–	–	TCA	5,184	5,257	74	–	–	TCA
tRNA-Ala	L	5,103	5,171	69	–	–	TGC	5,259	5,327	69	–	–	TGC
tRNA-Asn	L	5,174	5,246	73	–	–	GTT	5,330	5,402	73	–	–	GTT
tRNA-Cys	L	5,247	5,313	67	–	–	GCA	5,405	5,471	67	–	–	GCA
tRNA-Tyr	L	5,313	5,383	71	–	–	GTA	5,472	5,541	70	–	–	GTA
COI	H	5,385	6,935	1,551	GTG	AGG	–	5,543	7,093	1,551	ATG	AGG	–
tRNA-Ser	L	6,927	7,000	74	–	–	TGA	7,085	7,158	74	–	–	TGA
tRNA-Asp	H	7,005	7,073	69	–	–	GTC	7,163	7,231	69	–	–	GTC
COII	H	7,076	7,759	684	ATG	TAA	–	7,234	7,917	684	ATG	TAA	–
tRNA-Lys	H	7,761	7,830	70	–	–	TTT	7,919	7,989	71	–	–	TTT
ATPase 8	H	7,832	7,999	168	ATG	TAA	–	7,991	8,158	168	ATG	TAA	–
ATPase 6	H	7,990	8,673	684	ATG	TAA	–	8,149	8,832	684	ATG	TAA	–
COIII	H	8,673	9,456	784	ATG	T–	–	8,832	9,615	784	ATG	T–	–
tRNA-Gly	H	9,457	9,525	69	–	–	TCC	9,616	9,684	69	–	–	TCC
ND3	H	9,526	9,877	352	ATC	TAA	–	9,685	10,036	352	ATA	TAA	–
tRNA-Arg	H	9,882	9,950	69	–	–	TCG	10,041	10,109	69	–	–	TCG
ND4L	H	9,952	10,248	297	ATG	TAA	–	10,111	10,407	297	ATG	TAA	–
ND4	H	10,242	11,619	1,378	ATG	T–	–	10,401	11,778	1,378	ATG	T–	–
tRNA-His	H	11,620	11,689	70	–	–	GTG	11,779	11,848	70	–	–	GTG
tRNA-Ser	H	11,690	11,755	66	–	–	GCT	11,849	11,914	66	–	–	GCT
tRNA-Leu	H	11,756	11,826	71	–	–	TAG	11,915	11,985	71	–	–	TAG
ND5	H	11,827	13,644	1,818	GTG	TAG	–	11,986	13,815	1,830	ATG	TAA	–
Cytb	H	13,655	14,797	1,143	ATG	TAA	–	13,815	14,957	1,143	ATG	TAA	–

Feature	Strand	Brahminy kite (*Haliastur indus*)						Shikra (*Tachyspiza badius poliopsis*)					
		Position		Length	Start	Stop	Anticodon	Position		Length	Start	Stop	Anticodon
		From	to	(bp)	codons	codons		From	to	(bp)	codons	codons	
tRNA-Thr	H	14,800	14,869	70	–	–	TGT	14,960	15,029	70	–	–	TGT
Control region	H	14,870	16,402	1,533	–	–	–	15,509	16,768	1,260	–	–	–
tRNA-Pro	L	16,403	16,472	70	–	–	TGG	16,769	16,838	70	–	–	TGG
ND6	L	16,480	16,998	519	ATG	TAG	–	16,850	17,368	519	ATG	TAA	–
tRNA-Glu	L	17,002	17,072	71	–	–	TTC	17,372	17,442	71	–	–	TTC
Pseudo-control region	H	17,073	19,986	2,914	–	–	–	17,443	143	652	–	–	–

Table S3. Translational efficiency of mitogenomes in the family Accipitridae

Subfamily	Species	Mitochondrial oxidative phosphorylation (OXPHOS) genes												
		Complex I							Complex III	Complex IV			Complex V	
		ND1	ND2	ND3	ND4	ND4L	ND5	ND6	CYTB	COI	COII	COIII	ATP6	ATP8
Buteoninae	*Buteo hemilasius*	0.50	0.53	0.61	0.49	0.34	0.46	0.35	0.52	0.54	0.49	0.52	0.56	0.45
	Buteo buteo	0.52	0.54	0.62	0.52	0.44	0.49	0.41	0.51	0.53	0.49	0.53	0.57	0.41
	Buteo lagopus	0.47	0.51	0.59	0.49	0.34	0.49	0.31	0.56	0.53	0.49	0.49	0.57	0.41
	Butastur indicus	0.54	0.42	0.56	0.45	0.55	0.53	0.33	0.46	0.48	0.56	0.50	0.44	0.46
	Butastur liventer	0.49	0.46	0.58	0.47	0.41	0.58	0.27	0.49	0.55	0.61	0.50	0.45	0.42
	Haliaeetus albicilla	0.46	0.50	0.48	0.40	0.37	0.52	0.35	0.43	0.49	0.55	0.50	0.48	0.34
	Haliastur indus	0.49	0.49	0.52	0.42	0.51	0.44	0.35	0.49	0.54	0.48	0.53	0.49	0.46
	Milvus migrans	0.58	0.53	0.57	0.43	0.54	0.49	0.49	0.48	0.56	0.53	0.48	0.52	0.57
Circinae	*Circus spilonotus spilonotus*	0.47	0.57	0.55	0.39	0.56	0.42	0.42	0.46	0.51	0.53	0.46	0.43	0.17
	Circus melanoleucos	0.48	0.60	0.57	0.41	0.53	0.44	0.36	0.46	0.55	0.46	0.50	0.42	0.22
	Circus cyaneus	0.48	0.47	0.40	0.48	0.44	0.45	0.34	0.50	0.56	0.58	0.53	0.44	0.43
	Circus teauteensis	0.46	0.41	0.44	0.46	0.59	0.47	0.36	0.55	0.56	0.55	0.44	0.49	0.39
Accipitrinae	*Accipiter gentilis* (*Astur gentilis*)	0.45	0.50	0.45	0.51	0.41	0.52	0.41	0.46	0.50	0.47	0.51	0.46	0.37
	Accipiter nisus	0.48	0.56	0.52	0.52	0.58	0.49	0.51	0.48	0.52	0.57	0.47	0.60	0.55
	Accipiter soloensis (*Tachyspiza soloensis*)	0.49	0.49	0.53	0.43	0.49	0.48	0.32	0.41	0.55	0.53	0.49	0.59	0.63
	Accipiter badius poliopsis (*Tachyspiza badius poliopsis*)	0.53	0.62	0.62	0.46	0.50	0.51	0.55	0.50	0.53	0.60	0.43	0.43	0.50
	Accipiter gularis iwasakii (*Tachyspiza gularis iwasakii*)	0.55	0.60	0.36	0.48	0.54	0.47	0.31	0.49	0.49	0.57	0.45	0.34	0.66
	Accipiter virgatus (*Tachyspiza virgatus*)	0.54	0.60	0.34	0.48	0.54	0.47	0.31	0.48	0.49	0.57	0.43	0.34	0.66
	Accipiter trivirgatus (*Lophospiza trivirgatus*)	0.51	0.53	0.59	0.47	0.63	0.45	0.40	0.50	0.49	0.59	0.58	0.41	0.85
Aquilinae	*Aquila nipalensis*	0.46	0.49	0.45	0.49	0.36	0.43	0.27	0.50	0.56	0.50	0.55	0.48	0.74
Aegypiinae	*Gyps fulvus*	0.52	0.47	0.35	0.47	0.52	0.49	0.57	0.48	0.59	0.53	0.62	0.46	0.56
Circaetinae	*Circaetus pectoralis*	0.51	0.54	0.46	0.49	0.50	0.49	0.45	0.46	0.56	0.52	0.47	0.51	0.55
Perninae	*Pernis ptilorhynchus orientalis*	0.44	0.61	0.61	0.46	0.43	0.44	0.47	0.48	0.62	0.51	0.50	0.46	0.68
Elaninae	*Elanus caeruleus*	0.44	0.47	0.40	0.51	0.32	0.43	0.46	0.50	0.48	0.47	0.46	0.46	0.33
